# Efficient removal of drug-resistant *Providencia alcalifaciens* and its associated QnrS2 antibiotic-resistance genes by carbon-doped polymer carbon nitride

**DOI:** 10.1039/d5ra09506a

**Published:** 2026-01-22

**Authors:** Mingao Li, Jingshuang Zhang, Gengyuan Zhang, Yidan Zhang, Jinqiao Li, Li Wang, Guangbo Che, Qianyu Liu, Xiaoteng Liu

**Affiliations:** a Science and Technology Innovation Center of Jilin Province for Targeted Identification and Photocatalytic Degradation Materials, College of Engineering, Jilin Normal University Siping 136000 China; b Key Laboratory of Preparation and Application of Environmental Friendly Materials of the Ministry of Education, College of Chemistry, Jilin Normal University Changchun 130103 China; c Jilin Provincial Key Laboratory of Western Jilin's Clean Energy, Baicheng Normal University Baicheng 137000 China guangboche@bcnu.edu.cn; d College of Mathematics and Computer, Jilin Normal University Siping 136000 China liuqianyu0809433@126.com; e School of Engineering, Physics and Mathematics, Northumbria University Newcastle Upon Tyne NE1 8ST UK Terence.liu@northumbria.ac.uk

## Abstract

Pathogenic bacteria posed a serious threat to water ecosystems and might even have triggered disease outbreaks. In this study, a carbon-doped polymer carbon nitride (C-PCN) composed of numerous interwoven and stacked ultrathin lamellar units was fabricated *via* a simple stepwise calcination strategy. Compared with the polymer carbon nitride (PCN), C-PCN exhibited more remarkable photocatalytic performance for the *Providencia alcalifaciens* (*P. alcalifaciens*) isolated from a local hospital's waste water. C-PCN with a concentration of 0.4 mg mL^−1^ killed 7.07 log *P. alcalifaciens* within 100 min, whereas PCN could only inactivate 2.38 log *P. alcaliphilus* under the same conditions. Moreover, C-PCN could remove 99.87% antibiotic-resistance genes (ARGs) QnrS2 within 6 h. We addressed the gap in the existing research on inactivated *P. alcalifaciens*, and the fragmentation pathway of circular plasmids during photocatalysis reaction was observed *via* atomic force microscopy (AFM). The incorporation of carbon enhanced the visible light absorption capability of C-PCN and promoted more efficient charge separation. Mechanism investigation revealed that ˙O_2_^−^ and ˙OH were the vital reactive oxygen species (ROS) for antibiotic-resistance bacteria (ARB) inactivation and ARG degradation. ROS could induce cell rupture by damaging cellular membranes and disrupt metabolic processes by affecting enzyme activity. Additionally, a small-scale continuous-flow device could inactivate bacteria in hospital wastewater in 2.5 h under natural light irradiation, thus laying a foundation for advanced hospital wastewater treatment.

## Abstract 2

Pathogenic bacteria posed a serious threat to water ecosystems and might even have triggered disease outbreaks.

## Graphical abstract

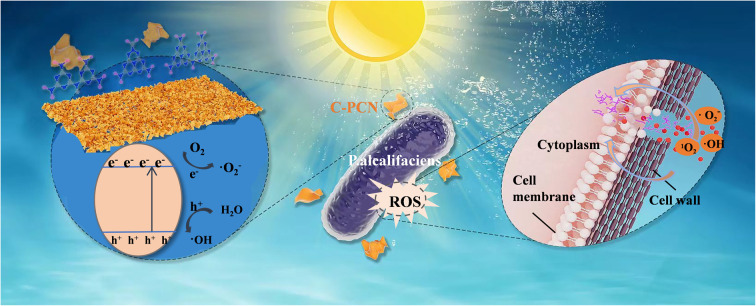

## 1 Introduction

*Providencia alcalifaciens* (*P. alcalifaciens*) is a conditionally pathogenic bacterium within the family Enterobacteriaceae.^1,2^ It is predominantly found in natural environments, including soil and aquatic systems, as well as in the gastrointestinal tracts of humans and animals.^3–5^ This organism can gain entry into the human body through ingestion of contaminated food or water or *via* direct contact, leading to opportunistic infections such as gastroenteritis and urinary tract infections.^6^ Especially in hospital settings, the emergence of drug-resistant strains had significantly increased the difficulty of clinical prevention and control.^7,8^

Consequently, there arose an urgent requirement to develop green and sustainable tactics for efficiently getting rid of antibiotic-resistance bacteria (ARB) and antibiotic-resistance genes (ARGs).^9,10^ Among numerous antibacterial technologies (*e.g.*, chlorination, ozonation, and ultraviolet irradiation), photocatalytic antibacterial technology stood out owing to its distinctive advantages.^11^ This technology enabled rapid and efficient bacterial inactivation without inducing evident drug resistance, and was thus recognized as a technology driven by green and renewable energy.^12,13^ Over the past several decades, researchers had developed a wide range of visible-light-responsive photocatalysts, encompassing silver-based materials, bismuth-based materials, sulfides, organic semiconductors, and oxides.^14^ However, the majority of them were subjected to certain limitations: photocatalysts had low visible-light utilization efficiency and suffered from e^−^/h^+^ recombination.^15,16^

Polymerized carbon nitride (PCN), an emerging metal-free photocatalyst, exhibited an appropriate band gap of roughly 2.7 eV, along with high chemical stability, low synthesis cost, and excellent biocompatibility.^17,18^ As an environmentally benign material, PCN demonstrated significant potential in antibacterial applications.^19^ Numerous studies had demonstrated that PCN and its derived composite materials could effectively inactivate common pathogenic bacteria, including *Escherichia coli* and *Staphylococcus aureus*, under visible light irradiation.^20,21^ However, to date, no reports had documented its efficacy in inactivating *P. alcalifaciens*. To address the aforementioned research gap, wastewater samples were collected from a local hospital, and *P. alcalifaciens* was successfully isolated.

Herein, a stepwise calcination strategy was proposed for the synthesis of carbon-doped polymerized carbon nitride (C-PCN). Owing to the optimized structure of the as-synthesized C-PCN, the charge migration and separation efficiency of this material were significantly promoted, thereby remarkably enhancing its photocatalytic activity. Under identical visible light irradiation conditions, its photocatalytic antibacterial efficiency was significantly higher than that of pristine nanosheet-structured carbon nitride. This study systematically investigated the influence of various factors on photocatalytic antibacterial performance and antibiotic resistance gene removal efficiency. The primary active species, their generation pathways, and the mechanisms of bacterial inactivation were further elucidated. Furthermore, a small-scale flow-through sewage treatment device was designed to achieve continuous inactivation of multidrug-resistant *P. alcalifaciens* under natural light, thereby laying a foundation for the advanced treatment of hospital wastewater.

## 2 Experimental section

### 2.1 Synthesis of PCN and C-PCN

All chemicals employed in this study were of analytical grade, as specified in Text S1. No further purification of the reagents was performed.

Synthesis of the catalysts, all characterizations and the detailed specifications of the instruments employed in this study were provided in Text S2.

### 2.2 Isolation and antimicrobial susceptibility testing of *P. alcalifaciens*

The domestic sewage from a hospital in Siping City was collected and stored at 4 °C prior to use. A volume of 100 µL of wastewater was spread onto nutrient agar solid medium and incubated at 37 °C for 24 h. Colonies exhibiting a yellowish color and slightly translucent appearance were selected and subcultured onto fresh nutrient agar plates until morphologically uniform colonies were obtained. The isolated colonies were subjected to repeated purification cycles to obtain consistent colonies, which were identified as *P. alcalifaciens*, for detailed information, referring to Table S1. Morphological and structural characteristics of the isolated strain, including optical microscopy images and colony morphology (Fig. S1a and b), and SEM images (Fig. S1c and d). When the antibiotic resistance of *P. alcalifaciens* was detected through the Kirby–Bauer (K–B) disk diffusion method, the results indicated that *P. alcalifaciens* was resistant to ampicillin, cefathiamidine, cefuroxime, midecamycin, piperacillin, gentamicin, oxacillin, penicillin G, spectinomycin, clarithromycin, vancomycin. For detailed information, please refer to Text S3 and Fig. S2.

### 2.3 Photocatalytic disinfection under visible light irradiation

The experimental setup included both dark control (with catalyst, no light) and light control (without catalyst, with light). All experiments were performed in triplicate and detailed information was presented in Text S4.

To visualize the process of bacterial apoptosis more directly, the changes of *P. alcalifaciens* at different stages before and after the photocatalytic reaction were further investigated using Calcein/PI Bacterial Viability and SEM, detailed information was presented in Text S5 and S6. For bacterial recovery and cyclic stability assessments, detailed information was presented in Text S7 and S8.

### 2.4 Photocatalytic bacterial inactivation mechanism

The damage inflicted on bacteria by reactive species generated during the photocatalytic reaction was investigated using an experimental system. Detailed information integrity of the bacterial cell membrane was assessed through K^+^ leakage and protein leakage (Text S9), malondialdehyde (MDA) content (Text S10), and alkaline phosphatase (AKP) synthesis capacity assays (Text S11),^22,23^ were provided in SI file.

### 2.5 Removal of antibiotic resistance genes

Both the 16S rRNA gene and the resistance gene QnrS2 were used as reference genes to evaluate the ability of photocatalyst to effectively remove genes.^24^ The detailed information of polymerase chain reaction (PCR) amplification conditions and gel electrophoresis experiments were provided in Text S12.

To quantify the release and degradation of antibiotic resistance genes (ARGs) during photocatalytic inactivation, intracellular ARGs (iARGs) and extracellular ARGs (eARGs) were separately extracted and subsequently quantified using quantitative polymerase chain reaction (qPCR).^25^ Detailed information regarding the qPCR protocol (Text S13), primer sequences of QnrS2 (Tables S2), quantitative polymerase chain reaction (qPCR) reaction system (Tables S3), absolute quantification standard curve concentration of qPCR for QnrS2 gene (Tables S4) and standard curve (Fig. S3) were provided in SI file. The surface morphology of plasmids following photocatalytic degradation was examined using AFM,^26^ detailed information was documented in Text S14.

### 2.6 Analysis of photogenerated carrier behavior

Detailed information of electrochemical measurements, including photocurrent response and EIS, were documented in Text S15.

### 2.7 Exploration of practical applications

The cytotoxicity was detected by co-culturing NIH/3T3 with different concentrations of C-PCN,^27^ (Suzhou Starfish Biotechnology Co., Ltd, TCM-C752), detailed information was provided in Text S16.

To evaluate the potential practical applicability of C-PCN, a small-scale continuous-flow wastewater treatment system was designed to achieve continuous inactivation of multi-drug-resistant *P. alcalifaciens* under natural light irradiation,^28^ detailed information was provided in Text S17.

## 3 Results and discussion

### 3.1 Morphological and chemical characteristics of PCN and C-PCN

**Fig. 1:**
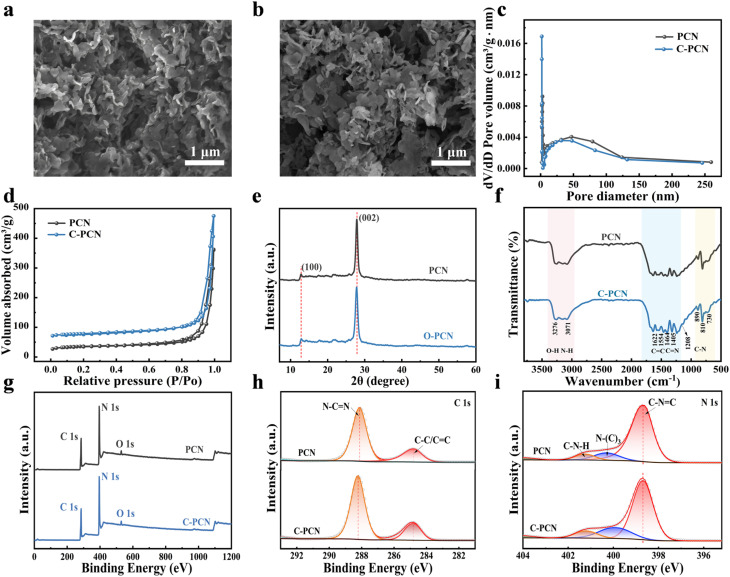
(a) SEM image of PCN, (b) SEM image of C-PCN, (c) N_2_ adsorption and desorption isotherms of PCN and C-PCN, (d) pore size distributions of PCN and C-PCN, (e) XRD images of PCN and C-PCN, (f) FT-IR spectra of PCN and C-PCN, (g) XPS survey of PCN and C-PCN, (h) high-resolution spectra of C 1s, (i) high-resolution spectra of N 1s.

SEM images of PCN and C-PCN were shown in Fig. 1a and b, and the elemental mapping of C-PCN was presented in Fig. S4, the uniform distribution of C and N elements has been observed. The PCN and C-PCN showed fluffy and soft agglomerates. In particular, C-PCN was composed of numerous ultrathin sheet-like units that interweaved and stacked with each other, forming an aggregate. Some of the edges of these sheet-like units exhibit an irregular shape. Fig. 1c and d presented the N_2_ adsorption and desorption isotherms of PCN and C-PCN, along with the corresponding pore size distributions. The specific surface area of PCN and C-PCN materials were 79.9 and 84.1 m^2^ g^−1^, respectively (Table S5). Compared to the original PCN nanosheets, the specific surface area of the C-PCN was a growth trend, which might provide more active sites for catalytic reactions and further explain the enhanced photocatalytic performance of C-PCN relative to PCN. Furthermore, the average pore diameters of PCN and C-PCN were positively correlated with their respective specific surface area. The average pore diameter of PCN was 23.2 nm, while that of C-PCN was 26.9 nm. To sum up, an ultrathin carbon nitride with a large specific surface area was successfully synthesized.

Next, XRD, FT-IR and XPS were used to characterize PCN and C-PCN structures. Two characteristic diffraction peaks were detected in the XRD patterns of PCN and C-PCN (Fig. 1e), corresponding to the (100) and (002) planes of the heptazine-based structure. The FT-IR spectra of PCN and C-PCN were presented in Fig. 1f. All samples displayed three prominent absorption bands at 810 cm^−1^, 1200–1700 cm^−1^, and 3000–3700 cm^−1^, which were characteristic of carbon nitride and could be attributed to the bending vibration of the triazine ring, the stretching vibrations of C=N and C–N bonds, terminal amino groups on the framework, and surface-adsorbed –NH and O–H groups,^29,30^ respectively.

The bonding states of the elements in the sample were further investigated using XPS. The XPS survey spectrum (Fig. 1g) confirmed the presence of three elements in the sample, including C, N, and O. The primary C 1s peak at 288.2 eV was attributed to sp^2^-hybridized carbon (N–C=N) within the nitrogen-containing aromatic ring. A secondary weak peak at a binding energy of 284.8 eV could be assigned to impurity carbon species, including C–C/C=C moieties.^31^ The XPS peak deconvolution of the C 1s spectrum (Fig. 1h) clearly revealed differences in carbon chemical states between PCN and C-PCN. Carbon doping modified the local bonding environment and alters the relative distribution of carbon species. The altered carbon configuration following C-PCN functionalization contributed to enhanced photocatalytic performance, which could be attributed to improved charge carrier separation induced by oxygen species and increased surface reactivity. The N 1s spectrum in Fig. 1i could be deconvoluted into three components at 398.8 eV, 400.1 eV and 401.3 eV. The peak at 398.9 eV was assigned to C–N=C bonding, while the peak at 400.1 eV corresponded to N–(C)_3_ species, while the peak at 401.3 eV corresponded the N atoms in the partially polymerized C–N–H structure respectively, and the action of the N–H side group.^32^ The results of elemental analysis demonstrated that after treatment at temperatures above 600 °C, the C/N atomic ratio of carbon nitride increased, change from 0.94 to 0.98, gradually approaching the 1 : 0 ratio of graphite, detailed information was available at Table S6. This variation was attributed to the thermal desorption of nitrogen-containing groups (*e.g.*, amino and cyan groups) in carbon nitride under high-temperature conditions, which caused the loss of nitrogen; in contrast, the carbon skeleton was relatively preserved and underwent gradual restructuring, ultimately resulting in an elevated proportion of carbon.^33,34^

### 3.2 Photoelectrochemical properties of PCN and C-PCN

**Fig. 2:**
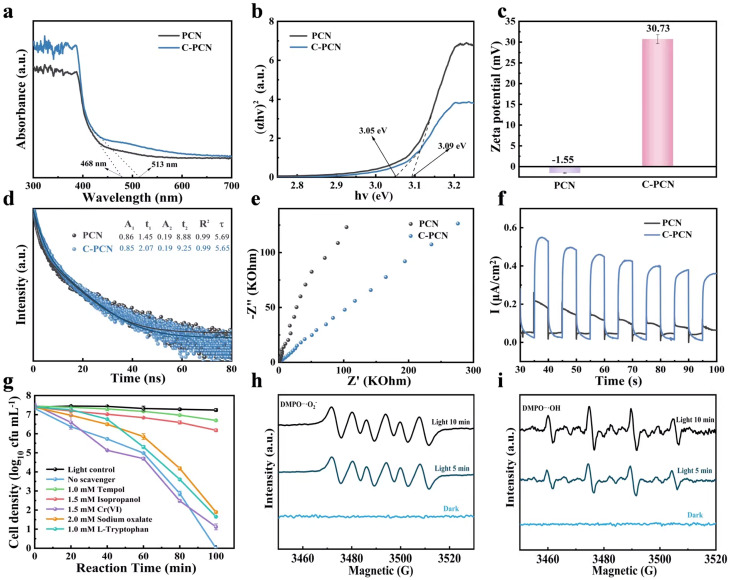
(a) UV-Vis DRS, (b) Tauc line, (c) zeta tests of the PCN and C-PCN, (d) time-resolved photoluminescence decay spectra (TRPL), (e) EIS plot and (f) the periodic photocurrent of PCN and C-PCN samples; (g) impacts of various active species on *P. alcalifaciens* inactivation under visible light illumination, (h) the electron paramagnetic resonance spectrum of ˙O_2_^−^ obtained in the DMPO solution, (i) the EPR spectrum of ˙OH obtained in an aqueous medium.

To research the optical properties of PCN and C-PCN, UV-Vis DRS was performed on the samples, as illustrated in Fig. 2a. Compared with PCN, C-PCN demonstrated significantly enhanced absorbance in the ultraviolet region (200–400 nm) and a portion of the visible light region (*e.g.*, 400–500 nm), indicating its stronger capacity to absorb shorter wavelength light, predominantly in the ultraviolet range. This phenomenon could be attributed to the modification of the electronic structure induced by carbon doping, which extends the light absorption range. The incorporation of carbon might enhance the capacity of material to absorb ultraviolet and visible light, thereby improving its photoelectronic and photocatalytic performance. As illustrated in Fig. 2b, the band gap was determined through linear extrapolation to the point of intersection with the *x*-axis. The results showed that the band gap of PCN was approximately 3.09 eV, whereas that of C-PCN was about 3.05 eV. This indicated a slight reduction in the band gap following carbon doping, accompanied by a redshift in the absorption edge. Such a change might have enhanced the ability of the material to absorb visible light, thereby promoting the generation of photogenerated charge carriers, improving charge separation efficiency, and ultimately contributing to enhanced photocatalytic antibacterial performance.^35^ The excitonic processes of PCN and C-PCN were investigated using steady-state photoluminescence (PL) measurements (Fig. S5). PCN exhibited a strong emission peak at 454 nm, which was attributed to the π → π* electronic transition; this observation indicated a high charge recombination rate of PCN. The C-PCN sample exhibited two fluorescence emission peaks at 454 nm and 478 nm, which were respectively attributed to the π → π* and *n* → π* electronic transitions. The fluorescence intensity of C-PCN was significantly reduced, thereby substantially suppressing electron–hole recombination. In C-PCN, the reduction in bulk and in-plane diffusion lengths for charge carrier transport enabled the rapid migration of photoexcited charge carriers, further confirming the excellent charge separation efficiency of C-PCN. The structure of C-PCN with reduced bulk and in-plane diffusion lengths for charge carrier transport enabled the rapid transfer of photoexcited charge carriers. In C-PCN, carbon doping significantly enhances charge separation efficiency. The incorporation of intermediate bandgap states through doping effectively suppresses electron–hole recombination and facilitates charge transfer, thereby improving photoresponse and carrier mobility.^36–38^

The zeta potential of the PCN and C-PCN was measured (Fig. 2c), revealing values of −1.55 mV for PCN and +30.73 mV for C-PCN, respectively. This significant difference reflected distinct surface chemical properties between the two materials. The fundamental structural unit of PCN-tris-*s*-triazine-is nitrogen-rich and typically featured abundant functional groups such as amino (–NH_2_) groups, along with an extended π-electron conjugated system. In near-neutral aqueous environments, these surface groups may have undergone protonation or deprotonation, resulting in a slightly negative surface charge on pristine PCN. Partial substitution of nitrogen atoms with carbon atoms altered the electron density distribution and surface charge characteristics. The observed reversal of the zeta potential from negative to positive provided direct evidence that carbon doping effectively modified the electronic structure and surface chemistry of PCN, leading to a fundamental transformation in its surface charge behavior.^39,40^

Time-resolved photoluminescence (TR-PL) spectra demonstrated that the average fluorescence lifetimes of PCN and C-PCN were 5.69 ns and 5.65 ns, respectively (Fig. 2d).

EIS was closely associated with photocatalytic performance and provided insights into the influence of material structure on surface charge transfer kinetics. The EIS Nyquist plot exhibited a semicircular arc, the radius of which reflected the charge transfer resistance within the photocatalytic material. A smaller radius corresponded to lower resistance and indicated more efficient charge transfer. As illustrated in Fig. 2e, C-PCN displayed the smallest arc under light irradiation, suggesting the most efficient separation of electron–hole pairs and thereby confirming its superior interfacial charge transfer capability during the photocatalytic process. The transient photocurrent of C-PCN under visible-light irradiation (*λ* > 420 nm) was substantially stronger than that of PCN (Fig. 2f), it had been demonstrated that the effective separation of electron–hole pairs occurred in the C-PCN structure. Moreover, the structure achieved considerably enhanced the inherently sluggish charge mobility of pristine PCN. The transient photocurrent response and EIS results demonstrated that C-PCN facilitated a more efficient separation of electron–hole pairs and enhanced the transfer of photogenerated charge carriers.

The oxidizing species directly generated during the photocatalytic reaction included holes (h^+^) and electrons (e^−^), while those indirectly produced included hydroxyl radicals (˙OH), superoxide radicals (˙O_2_^−^), and singlet oxygen (^1^O_2_). These reactive species were among the most commonly involved in photocatalytic antibacterial applications. Radical scavenging experiments could be employed to identify the key species that played a dominant role in the photocatalytic process. As illustrated in Fig. S6, we selected the optimal concentration of scavengers to conduct the active substance capture experiments to ensure that all types of free radicals could be completely captured. The cytotoxicity of various scavenging agents toward bacteria was evaluated. The results indicated that individual scavengers did not affect bacterial activity under light irradiation.

In this study, Tempol (1.0 mM) for scavenging ˙O_2_^−^, isopropanol (0.5 mM) for scavenging ˙OH, sodium oxalate (2.0 mM) for scavenging h^+^, l-tryptophan (1.0 mM) for scavenging ^1^O_2_ and Cr(vi) (0.1 mM) for scavenging e^−^.^41^

However, upon the addition of different scavenging agents, the antibacterial efficacy of C-PCN against *P. alcalifaciens* exhibited varying degrees of reduction (Fig. 2g). Therefore, it could be inferred that the ROS involved in the photocatalytic process followed the activity sequence ˙O_2_^−^ > ˙OH > h^+^ > ^1^O_2_ > e^−^. To further elucidate the photocatalytic reaction mechanism, electron spin resonance (EPR) analysis of active species was performed to detect ROS generation by C-PCN (Fig. 5h and i). Under dark conditions, no distinct EPR signals corresponding to ˙OH and ˙O_2_^−^ were detected, confirming the essential role of light irradiation in the generation of reactive species within the system. Under light conditions, the characteristic EPR peaks of these radicals exhibited significant fluctuations, indicating that these active species could be effectively excited and generated under visible light irradiation.

From this, it could be inferred that the mechanism of action was composed of three sequential steps: firstly, C-PCN was activated under visible light irradiation, resulting in the generation of photogenerated h^+^ and photogenerated e^−^. The unique ultrathin nanosheet structure of C-PCN facilitated efficient light absorption and charge carrier separation, thereby providing a structural and functional basis for subsequent antibacterial reactions. Subsequently, the photogenerated e^−^ react with molecular oxygen to generate ˙O_2_^−^, while the photogenerated h^+^ reacted with H_2_O or –OH to produce ˙OH.^42,43^ These active species possessed strong oxidative properties and served as the primary antibacterial agents. Ultimately, these active species disrupted the membrane structure of *P. alcalifaciens*, leading to the alterations of membrane permeability and the leakage of intracellular contents. ROS could damage DNA, essential enzymes, cellular proteins, and ribosomes. The consequent oxidative stress could further disrupt cell membranes, electron transport chains, and energy metabolism processes, induce the leakage of intracellular components, and ultimately lead to the death of bacterial cells.^44^

In summary, C-PCN generated charge carriers upon light irradiation, which were subsequently converted into oxidizing species. By utilizing light energy to drive redox reactions, it induced oxidative damage to bacterial cellular structures, resulting in the leakage of intracellular contents and thereby achieving a potent antibacterial effect.

### 3.3 Photocatalytic antibacterial performance

**Fig. 3:**
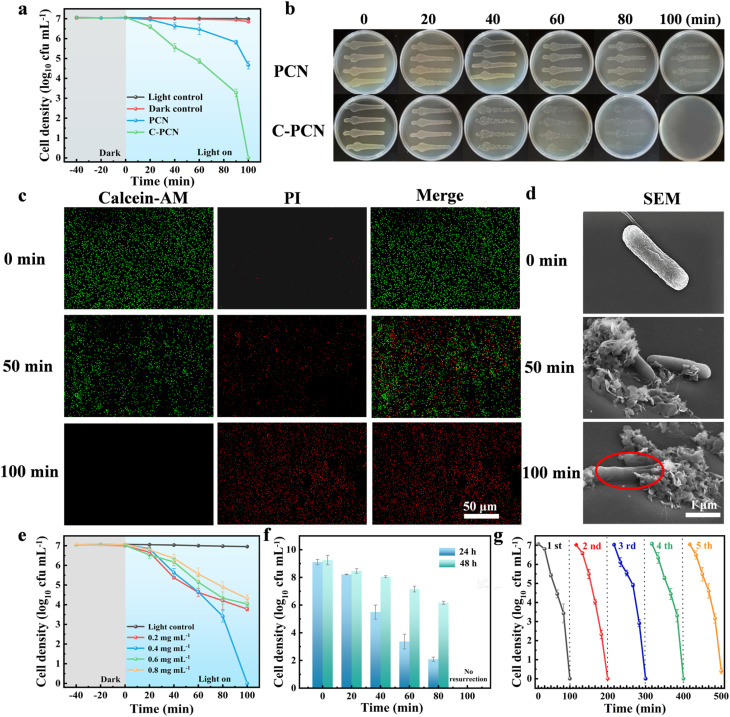
(a) The antibacterial effects of PCN and C-PCN under visible light irradiation for 100 min, (b) image of the colony plates during the reaction process, (c) fluorescence staining images during the antibacterial process, (d) SEM images during the antibacterial process, (e) inactivation efficiency of *P. alcalifaciens* by different concentrations of C-PCN under visible light irradiation within 100 min, (f) bacterial recovery experiment of *P. alcalifaciens*, (g) photocatalytic disinfection stability of C-PCN.

Fig. 3a showed the antibacterial effects of PCN and C-PCN at a concentration of 0.4 mg mL^−1^ under visible light irradiation for 100 min, as well as the corresponding photos of *P. alcalifaciens* during the antibacterial reaction (Fig. 3b), the colony images of the light control and dark control groups were presented in Fig. S7. The dark control (with catalyst, no light) and the light control (without catalyst, with light) showed minimal inactivation of *P. alcalifaciens*. Under visible light excitation, PCN demonstrated relatively low antibacterial efficiency, achieving only approximately 2.38 log inactivation of *P. alcalifaciens* after 100 min of irradiation. In contrast, C-PCN achieved a significantly higher inactivation level of approximately 7.07 log under the same experimental conditions. These results clearly demonstrated that C-PCN exhibited the highest antibacterial activity among the tested materials.

The overall antibacterial activity of the photocatalytic material was evaluated *via* Calcein/PI Bacterial Viability Kits. Using an inverted fluorescence microscope, the phenomenon of apoptosis in *P. alcalifaciens* could be clearly detected. The fluorescence of the active bacteria appeared green, whereas the fluorescence emitted by the dead bacteria appeared red. As presented in Fig. 3c, the initial green fluorescence exhibited a gradual weakening trend, while the red fluorescence showed a progressive increase.^45^ Following 100 min of treatment, all bacterial emitted red fluorescence, which indicated that the catalyst had achieved complete inactivation of *P. alcalifaciens* at this time point. And the ROS produced by *P. alcalifaciens* at different time intervals during the photocatalytic process were evaluated, for detailed information, please refer to Text S18 and Fig. S8.

We conducted zeta tests on the bacteria before and after the photocatalytic antibacterial process (Fig. S9). The results showed that the zeta potential of the bacteria after treatment increased from 8.98 mV to approximately 15.09 mV, indicating a significant reduction in the surface negative charge. This confirmed that the photocatalytic process effectively disrupted the bacterial cell membrane structure, resulting in changes or leakage of surface groups, which was consistent with the conclusion in the antibacterial mechanism that reactive oxygen species attack causes membrane damage.

For the purpose of further confirming the bacterial inactivation process, the structural and morphological changes of *P. alcalifaciens* in the course of its inactivation under visible light at various time intervals were observed using SEM.^46,47^ Taking C-PCN as a sample, at the initial stage of photocatalytic sterilization, *P. alcalifaciens* had a complete rod shape (Fig. 3d). With the visible light irradiation, slight deformation of *P. alcalifaciens* could be observed at 6 min, the bacteria had experienced significant deformation and displayed a concave configuration. When the light irradiation duration reached 100 min, the cell walls of *P. alcalifaciens* had been disrupted, leading to severe damage to the bacterial cells. Fig. 3e illustrated the antibacterial activity of *P. alcalifaciens* under visible light irradiation in the presence of varying concentrations of C-PCN (0.2, 0.4, 0.6, and 0.8 mg mL^−1^), together with the corresponding colony photographs (Fig. S10). The results indicated that after 100 min, the bacterial density decreased by 3.22 log, 2.98 log, and 2.76 log at concentrations of 0.2, 0.6, and 0.8 mg mL^−1^, respectively. The concentration of 0.4 mg mL^−1^ exhibited the highest photocatalytic antibacterial activity, resulting in a bacterial density reduction of 7.06 log. This could be attributed to the fact that insufficient photocatalyst cannot generate an effective antibacterial response, whereas an excessive amount might obstruct light penetration and consequently reduce photocatalytic efficiency.^48^ Therefore, a concentration of 0.4 mg mL^−1^ was optimal.

We measured the absorbance of the bacterial suspension at a wavelength of 600 nm during the antibacterial experiments with different catalyst concentrations (Fig. S11). We systematically investigated the variation of absorbance with catalyst concentration.

The results showed that as the catalyst concentration increased from 0.2 mg mL^−1^ to 0.8 mg mL^−1^, the absorbance of the solution gradually increased, indicating a decrease in transmittance. The antibacterial efficiency first enhanced and then was inhibited. This was attributed to changes in the utilization efficiency of light in the photocatalytic reaction. A concentration of 0.4 mg mL^−1^ was found to be optimal for antibacterial activity. Excessive catalyst could hinder active sites or block light, reduce light transmission and thus inhibit the photocatalytic reaction. We also measured the absorbance of suspensions with different concentrations.^49,50^

As illustrated in Fig. S12, the photocatalytic removal efficiency of varying bacterial loads under a fixed catalyst dosage was evaluated. At a catalyst dosage of 4 mg, all tested concentrations of *P. alcalifaciens* achieved optimal inactivation efficiency. As demonstrated in Fig. 3f, with the extension of photocatalytic reaction time, the number of regenerative bacteria gradually decreased, and drug-resistant bacteria were completely inactivated after 100 min. The corresponding agar plate photographs were presented in Fig. S13. This result demonstrated that the photocatalytic inactivation of *P. alcalifaciens* by C-PCN might have induced irreversible bacterial damage, thereby prevented self-repair and demonstrated significant advantages over other disinfection methods.

Three cycles of photocatalytic antibacterial experiments on *P. alcalifaciens* were conducted to study the performance stability of the C-PCN photocatalyst. The agar plate photos (Fig. S14), as shown in Fig. 3g, indicated that the C-PCN still maintained its high deactivation ability even after three cycles. The structural stability of the photocatalyst was analyzed through XRD (Fig. S15a), FT-IR (Fig. S15b), and XPS (Fig. S15c) characterization. The experimental results demonstrated that the structure of C-PCN remained almost unchanged before and after photocatalytic activity. The above experimental results demonstrated that the C-PCN photocatalyst was able to retain its intact structure and efficient photocatalytic activity even after undergoing multiple recycling cycles.

### 3.4 Mechanisms of bacterial inactivation

**Fig. 4:**
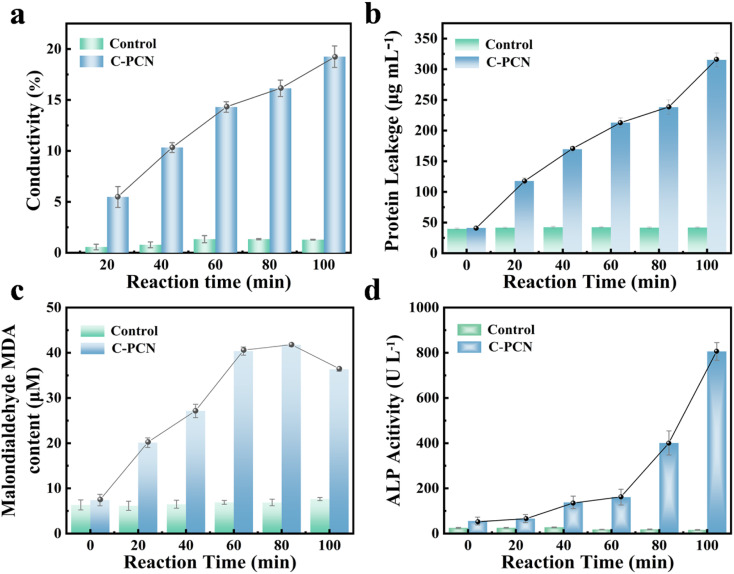
(a) Changes of protein leakage concentration of *P. alcalifaciens* treated with C-PCN, (b) changes of conductivity of *P. alcalifaciens* treated with C-PCN, (c) changes of MDA of *P. alcalifaciens* treated with C-PCN, (d) changes of AKP of *P. alcalifaciens* treated with C-PCN.

Due to the disruption of the cell membrane during the inactivation process, *P. alcalifaciens* exhibited leakage of inorganic salts. The antibacterial efficacy of C-PCN could be evaluated by determining the conductivity of the bacterial suspension under varying durations of light exposure. As shown in Fig. 4a, the conductivity of the solution gradually increases with prolonged C-PCN irradiation time. Compared with the 0 min time point, the conductivity of the bacterial suspension exhibited a 14.31% increase after 60 min and a 19.25% increase after 100 min. These results indicated that C-PCN was capable of generating reactive oxygen species (ROS) in the solution, which could attack bacterial and induce the leakage of inorganic salts. Since damage of the cell membrane could lead to an increase in the total protein content within the reaction solution, the antibacterial efficacy of C-PCN could be assessed by quantifying the amount of protein leaked into the bacterial suspension under varying durations of light exposure. A standard curve was established between the standard protein concentration and the optical density at 562 nm (Fig. S16), revealing that the mass of leaked protein increased with prolonged C-PCN irradiation time (Fig. 4b). Compared to the 0 min, the protein leakage from the bacterial suspension after 100 min of light exposure was approximately 6-fold higher than that of the light control group. The results demonstrated that the ROS generated by C-PCN were capable of attacking bacterial, compromising their structural integrity and inducing intracellular protein leakage. This observation aligned with the outcomes of the conductivity test.

MDA, the primary end product of lipid peroxidation, was capable of inducing cellular membrane damage. A standard calibration curve was established between the MDA concentration of the reference sample and the optical density at 532 nm (Fig. S17). The results revealed that the MDA content in bacterial increased significantly with prolonged photocatalytic treatment time (Fig. 4c), peaking at 41.76 µM, followed by a gradual decline in concentration. The reduction in MDA levels at later stages might have been attributed to its further oxidation by reactive species present in the photocatalytic reaction system. Upon exposure to antibacterial agents, bacterial might have initiated an oxidative stress response. The intracellular antioxidant defense system was activated as a protective response to mitigate oxidative stress; however, it might had been insufficient to fully neutralize the excessive ROS generated in the early stages. These ROS targeted the polyunsaturated fatty acids present on cell membrane, initiating lipid peroxidation reactions that subsequently led to a significant increase in MDA production. Bacteria possessed inherent mechanisms to repair damaged cell membranes and eliminate oxidative byproducts.^51^ Over time, these intracellular repair systems became increasingly effective, facilitating the restoration of membrane integrity and the removal of oxidative products such as MDA, thereby leading to a reduction in MDA levels.

To elucidate the inactivation mechanism of drug-resistant bacteria from an energetic perspective, variations in the concentration of AKP, also referred to as alkaline phosphomonoesterase and abbreviated as AKP, were monitored (Fig. 4d). A significant increase in AKP levels was observed within 100 min, indicating substantial cellular damage occurring during this time period. At 100 min, the AKP content reached its peak. As the reaction time progressed, a significant increase in AKP levels was observed, suggesting that the antibacterial agents might have compromised the bacterial cell membrane, causing the leakage of substances inside the cell components and disruption of ion homeostasis. AKP was closely associated with the structure and function of the cell membrane. The elevated levels of AKP might have reflected the adaptive response of the bacteria to repair the damaged membrane and preserve its structural integrity and physiological functionality.

We measured the zeta potential of the photocatalyst and bacterial cells (Fig. S18), and the results demonstrated that the C-PCN photocatalyst possessed a positive charge and thus exhibited a distinct adsorption capacity toward negatively charged bacteria. Given the limited diffusion range of free radicals, this close contact between the photocatalyst and bacteria effectively facilitated the direct interaction of free radicals with bacterial cells, thereby promoting membrane-disrupting reactions. The resultant oxidative stress further impaired bacterial cell membranes, electron transport chains, and energy metabolism pathways, induced the leakage of intracellular components and ultimately culminated in bacterial cell death.^52^

The antibacterial application mechanism of nanocomposites in addressing multi-drug resistance was illustrated as shown in the Fig. S19.

### 3.5 Degradation of ARGs during photocatalytic inactivation of *P. alcalifaciens*

**Fig. 5:**
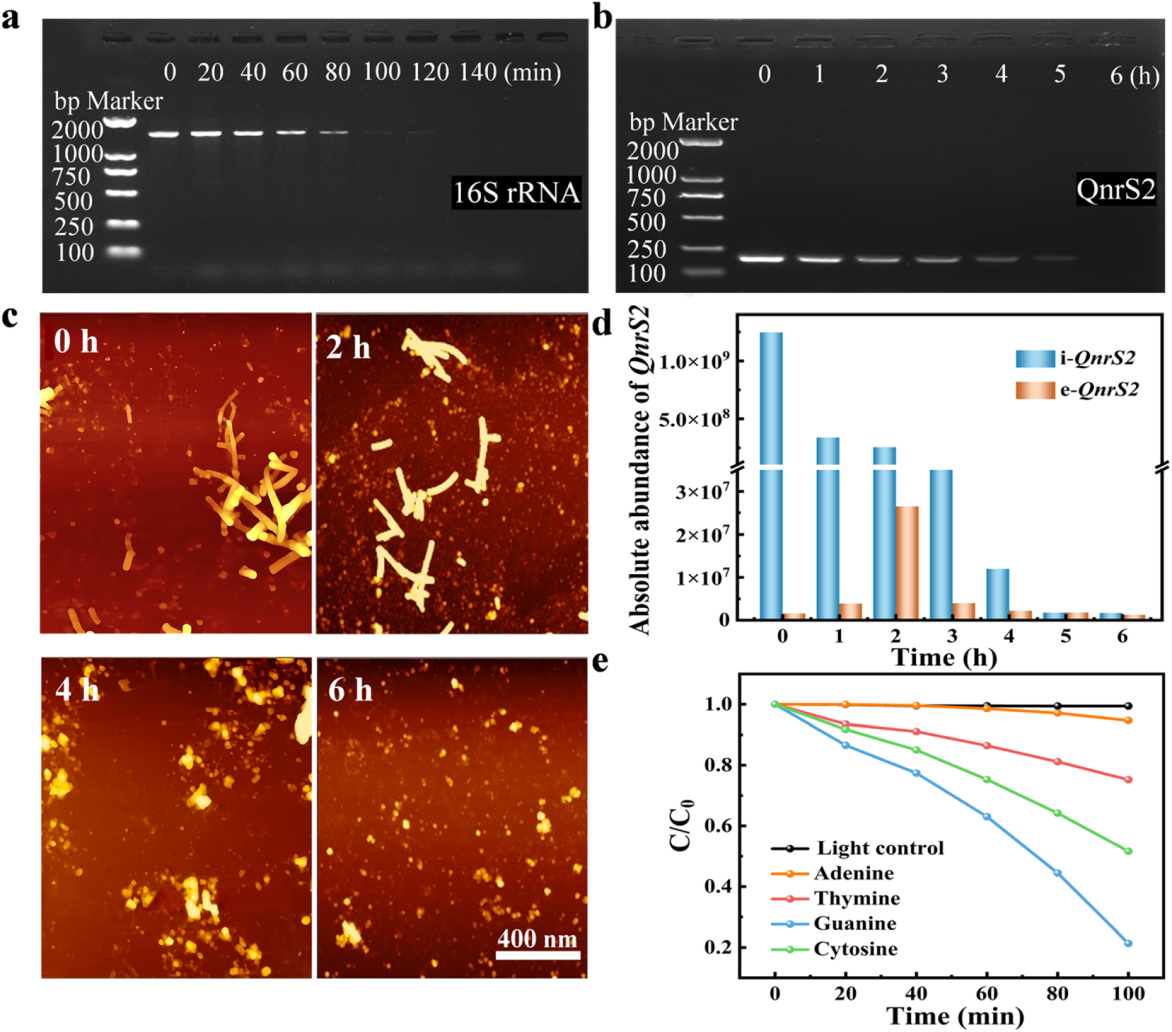
(a) DNA electrophoresis gel of 16S rRNA treated with C-PCN, (b) DNA electrophoresis gel of QnrS2 treated with C-PCN, (c) AFM images of the degradation process of DNA fragments, variations of QnrS2 ARGs at different photocatalytic inactivation times with the C-PCN, (d) trends in the variation of the relative abundances of the i-QnrS2 and e-QnrS2 genes, (e) base degradation experiment.

Fig. 5a illustrated the DNA damage in *P. alcalifaciens* during the photocatalytic antibacterial process, using 16S rRNA serving as the reference gene. The results showed that the 16S rRNA band was clearly visible at 0 min but had disappeared at 140 min. This suggested that the 16S rRNA gene had been damaged within 140 min during the photocatalytic disinfection process. Similarly, when QnrS2 was used as a target for the degradation of extracellular DNA, gel electrophoresis results in Fig. 5b displayed the QnrS2 band. This band gradually intensified from 0 to 6 h. This observation initially demonstrated that the QnrS2 had undergone degradation.^53,54^ As bacterial activity continued to decline, the QnrS2 gene was nearly completely degraded. The degradation behavior of plasmids during the photocatalytic inactivation process was investigated using AFM imaging. Prior to treatment, the plasmids exhibited large molecular structures. Morphological changes were observed at different treatment time points. Throughout the entire photocatalytic treatment process, the plasmid bands remained detectable. With the progression of the photocatalytic treatment time, the circular structure of the plasmid molecules was initially targeted (Fig. 5c). Subsequently, reactive species attacked the linear regions of the plasmid molecules, leading to a gradual reduction in their length. After 6 h of treatment, certain plasmids were ultimately fragmented into smaller pieces. During the photocatalytic inactivation process, both the structural integrity and surface morphology of the plasmid fragments, including ARGs, were compromised. The fragmentation pathway of the circular plasmid might represent a potential mechanism underlying ARG degradation. As shown in Fig. 5d, to evaluate the removal effect of C-PCN materials on ARG during the photocatalytic inactivation process, qPCR was used to quantitatively analyze the variations in the copy numbers of extracellular e-QnrS2 and intracellular i-QnrS2 resistance genes. Before processing, it was found that the relative abundance of the QnrS2 gene outside the cells was approximately 1 × 10^3^.^14^ It could have originated from the leakage of dead bacterial cells or the active release by the bacteria. The relative abundance of the e-QnrS2 gene exhibited a gradual increase within the first 0–2 h following photocatalytic inactivation. Subsequently, between 3 h and 6 h, a decrease in the relative abundance of the e-QnrS2 gene was observed. This trend might have been attributed to the initial active genetic transfer. Alterations in the antibacterial environment might have stimulated bacterial acquisition of resistance genes *via* horizontal gene transfer mechanisms, such as increased activity of mobile genetic elements and bacteriophages, thereby contributing to a rise in extracellular gene abundance. The later-stage horizontal gene transfer became restricted. As the antibacterial process progressed, the population of susceptible bacteria declined, along with a reduction in the number of bacteria capable of engaging in horizontal gene transfer. Concurrently, bacterial adaptation to the environment might have occurred, leading to a diminished necessity for genetic transfer. This resulted in a deceleration of the increasing trend in extracellular gene abundance, followed by a gradual decline. The continuous decline in the relative abundance of the intracellular i-QnrS2 gene confirmed that *P. alcalifaciens* had experienced a loss of membrane integrity, resulting in the release of the i-QnrS2 gene into the extracellular space. Fig. 5e showed the degradation of bases 0.4 mg mL^−1^ C-PCN could degrade approximately 80% of guanine within 100 min.

### 3.6 Practical application prospects

**Fig. 6:**
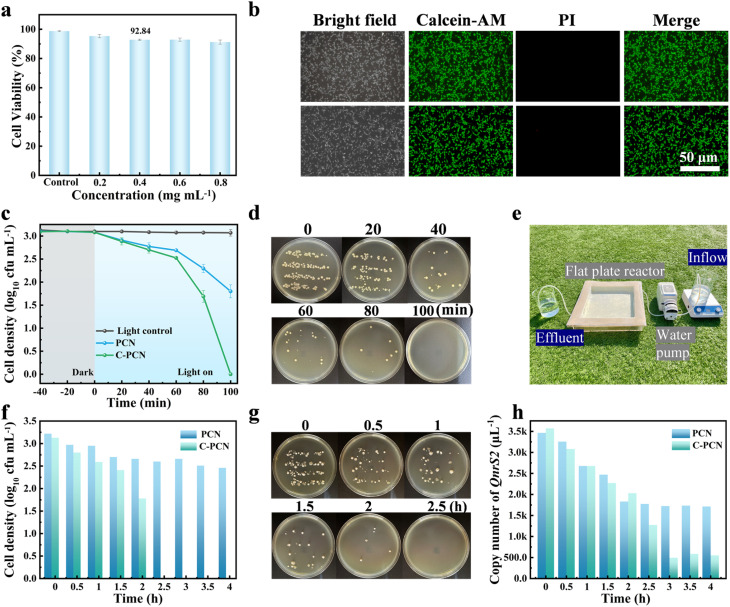
(a) The effects of different concentrations of C-PCN on the viability of NIH/3T3 cells, (b) fluorescence staining assessment of NIH/3T3 cell viability following varying durations of C-PCN, (c) the photocatalytic antibacterial properties of PCN and C-PCN in the hospital wastewater, (d) colony plate images; (e) continuous flowing water disinfection system device diagram, (f) the antibacterial performance removal effect of C-PCN in flowing water bodies under natural light exposure, (g) colony plate images, (h) the removal effect of the QnrS2 resistance gene.

To evaluate the biocompatibility of C-PCN, various concentrations of C-PCN solutions were co-cultured with NIH/3T3 mouse embryonic fibroblasts in a 37 °C cell incubator for 4 h, in order to assess the potential impact of this material on cell viability and proliferation.^55,56^ The influence of C-PCN on the proliferation of NIH/3T3 cells was determined *in vitro* by means of the Cell Counting Kit-8 (CCK-8), as illustrated in Fig. 6a. When the C-PCN concentration reached 0.4 mg mL^−1^, more than 92.84% of the cells remained viable under light exposure, suggesting that C-PCN exhibited no significant cytotoxic effects on NIH/3T3 cells. As illustrated in Fig. 6b, the fluorescence staining images of NIH/3T3 mouse embryonic fibroblasts in the control group exhibited a spindle-shaped or fusiform morphology.^57,58^ After the administration of 0.4 mg mL^−1^ C-PCN to the experimental group, no significant changes in cellular morphology were observed. Overall, no appreciable morphological differences were detected between experiments group and the control group. In summary, the results of the CCK-8 assay and the live/dead fluorescence imaging of NIH/3T3 cells collectively demonstrated that C-PCN exhibited minimal cytotoxicity and excellent biocompatibility under the antibacterial conditions employed in this study. These findings further supported the potential of C-PCN in real-application scenarios for the purpose of reducing the risk of pathogen transmission. To evaluate the disinfection capability of C-PCN in real water environments, Fig. 6c presented the photocatalytic antibacterial performance of C-PCN against hospital wastewater under varying durations of visible light irradiation, supported by agar plate photographs (Fig. 6d). The results demonstrated that C-PCN was capable of inactivating all bacteria present in the hospital wastewater within 100 min. Fig. 6e illustrated a small-scale mobile sewage treatment prototype designed to simulate the practical application scenario of C-PCN in wastewater treatment plants.^59^

Fig. 6f–h, respectively depicted the antibacterial efficacy of PCN and C-PCN photocatalysts at a concentration of 0.4 mg mL^−1^ in inactivating multidrug resistant *P. alcalifaciens* and the QnrS2 resistance gene under natural light irradiation. As shown in the agar plate photographs presented in Fig. 6g, the bacterial density in the C-PCN group was markedly lower than that in the PCN group, and complete bacterial inactivation was achieved after 2.5 h. Fig. 6h demonstrated that the copy number of the target gene in the C-PCN group decreased by 84.60% after 4 h, as determined by qPCR analysis. In summary, C-PCN exhibited effective disinfection performance in real water environments and could be immobilized onto non-woven fabric to construct a flat-plate reactor for solar-driven water disinfection.^60,61^ As a result, purified water with significantly reduced antibiotic resistance gene content could be obtained after a 4 h treatment period.

## 4 Conclusion

In summary, this study successfully synthesized carbon-doped polymeric carbon nitride (C-PCN) consisting of numerous interwoven and stacked ultrathin layers *via* a facile stepwise calcination approach. A highly pathogenic bacterium, *P. alcalifaciens*, was isolated from the wastewater of a local hospital. This organism had been associated with intestinal infections, urinary tract infections, and wound infections, among other clinical conditions. Given that C-PCN was capable of efficiently generating oxidizing species, a concentration of 0.4 mg mL^−1^ C-PCN was able to inactivate approximately 7.07 log units of *P. alcalifaciens* following 100 min of visible light irradiation. The antibacterial efficacy of C-PCN was approximately threefold higher than that of the original PCN. Furthermore, C-PCN exhibited high photostability, as no significant reduction in antibacterial activity was observed even after five consecutive reuse cycles. The results demonstrated that carbon incorporation enhanced the visible light absorption capacity of C-PCN, facilitated more efficient charge separation, increased the generation of reactive species, induced bacterial cell rupture *via* disrupting the cell membrane, and interfered with bacterial metabolic processes by altering enzyme activity. These effects collectively contributed to the improved efficacy of C-PCN in inactivating ARB and degrading ARGs. This study innovatively extracted the *P. alcalifaciens* from local hospital wastewater, filling the gap in the current research on photocatalytic inactivation of the *P. alcalifaciens*. Not only presented a novel strategy for enhancing the photocatalytic inactivation of ARB through solar energy utilization, but also demonstrated significant application potential in broader environmental remediation fields, offering an innovative approach to addressing ARB pollution in aquatic environments.

## Conflicts of interest

The authors declare that they have no known competing financial interests or personal relationships that could have appeared to influence the work reported in this paper.

## Supplementary Material

RA-016-D5RA09506A-s001

## Data Availability

Data will be made available on request. Supplementary information (SI) is available. See DOI: https://doi.org/10.1039/d5ra09506a.
