# Supplementary material for: Efficient removal of drug-resistant Providencia alcalifaciens and its associated QnrS2 antibiotic-resistance genes by carbon-doped polymer carbon nitride
Source: RSC Adv. 2026 Jan 22;16(6):4946–58. doi: 10.1039/d5ra09506a (PMC12826100; doi:10.1039/d5ra09506a)
Supplement: RA-016-D5RA09506A-s001 [file RA-016-D5RA09506A-s001.pdf]

Supplementary Information (SI) for RSC Advances.
This journal is © The Royal Society of Chemistry 2026

Supporting Information

Efficient removal of drug-resistant Providencia alcalifaciens and its associated QnrS2 antibiotic-resistance genes by carbon-doped polymer carbon nitride

Mingao Li [a], Jingshuang Zhang [a], Gengyuan Zhang [a], Yidan Zhang [b], Jinqiao Li [b], Li Wang [b], Guangbo Che [c,*], Qianyu Liu [d,*], Xiaoteng Liu[e,*]

[a] *Science and Technology Innovation Center of Jilin Province for Targeted Identification and Photocatalytic Degradation Materials, College of Engineering, Jilin Normal University, Siping, 136000, China*

[b] *Key Laboratory of Preparation and Application of Environmental Friendly Materials of the Ministry of Education, College of Chemistry, Jilin Normal University, Changchun, 130103, China.*

[c] *Jilin Provincial Key Laboratory of Western Jilin's Clean Energy, Baicheng Normal University, Baicheng, 137000, China*

[d] *College of Mathematics and Computer, Jilin Normal University, Siping, 136000, China*

[e] *School of Engineering, Physics and Mathematics, Northumbria University, Newcastle Upon Tyne, UK, NE1 8ST*

*Corresponding author: Guangbo Che (guangboche@bcnu.edu.cn), Qianyu Liu (liuqianyu0809433@126.com) and Xiaoteng Liu (Terence.liu@northumbria.ac.uk)

Content

Texts 3

Text S1. Chemical reagents 3

Text S2. Synthesis and characterization of the catalysts 4

Text S3. Detect the antibiotic resistance of *P. alcalifaciens* 5

Text S4. Photocatalytic disinfection under visible light irradiation 6

Text S5. Sample preparation for SEM analysis of *P.alcalifaciens* 7

Text S6. Sample Preparation for *P.alcalifaciens* Viability Assessment Using the Calcein/PI Assay 8

Text S7. Bacterial regrowth experiment 9

Text S8. cyclic stability assessments 10

Text S9. Protein leakage and conductivity assays 11

Text S10. Lipid oxidation detection 12

Text S11. Alkaline phosphatase activity assay 13

Text S12. PCR and gel electrophoresis experiments 14

Text S13. qPCR detection of target ARGs 15

Text S14. AFM image of plasmid 16

Text S15. Photoelectrochemical measurements 17

Text S16. Biological toxicity analysis 18

Text S17. Antibacterial for flowing water bodies 19

Text S18. ROS fluorescence detection 20

Figures 21

Fig. S1. Morphological and structural characteristics of *P. alcalifaciens* 21

Fig. S2. Select the optimal concentration of free radical scavenger. Antimicrobial susceptibility assay of *P. alcalifaciens* 22

Fig. S3. Absolute quantitative standard curve for QnrS2 gene qPCR. 23

Fig. S4. Elemental mapping of C-PCN 24

Fig. S5. PL spectra of PCN and C-PCN samples 25

Fig. S6. Select the optimal concentration of free radical scavenger 26

Fig. S7. The colony plates of photocatalytic disinfection experiments.................27
Fig. S8. ROS fluorescence detection..28
Fig. S9. Zeta tests of the *P. alcalifaciens*..29
Fig. S10. Antibacterial experiments with different catalyst concentrations. 30
Fig. S11. Absorbance experiments with different catalyst concentrations 31
Fig. S12. Antibacterial experiments under different bacterial concentrations. 32
Fig. S13. Photographs of bacterial agar plates for bacterial recovery..................33
Fig. S14. Antibacterial cyclic stability colony plate. 34
Fig. S15. Characterization of the cyclic stability of C-PCN 35
Fig. S16. Standard protein concentration vs. standard curve of OD562 nm.........36
Fig. S17. Standard MDA concentration vs. standard curve of OD532 nm...........37
Fig. S18. Zeta tests of the C-PCN and *P. alcalifaciens*. 38
Fig. S19. Antibacterial mechanism diagram 39
Tables 40
Table S1. Identification of strains 40
Table S2. Primer sequences of QnrS2..41
Table S3. FQ-PCR reaction system...42
Table S4. The qPCR standard curve of the QnrS2 gene. 43
Table S5. The BET of PCN and C-PCN 44
Table S6. Quantitative analysis of element content 45

Texts

Text S1 Chemical reagents

Urea was purchased from Sinopharm Chemical Regent. Phosphate buffer saline (PBS), nutrient agar, Tris Acetate-EDTA buffer (TAE), agarose and nutrient broth were acquired from Qingdao Hi-tech Industrial Park Hope Bio-technology. BCA Protein Assay Kit (BCA), Lipid Peroxidation MDA Assay Kit (MDA), Alkaline Phosphatase Assay Kit (AKP), Bacterial viability fluorescence assay kit was purchased from Ingenuity Life Technologies, Inc. Real-time quantitative PCR (qPCR) Mastermixes were purchased from QIAGEN Co, Ltd. Genomic DNA extraction kit (centrifugal column type), bacterial genomic DNA extraction kit (centrifugal column type), PCR Master Mix were purchased from TIANGEN BIOTECH (Beijing) Co., Ltd. All reagents used in this study were analytically pure. The ultrapure water used in the experiments was obtained from Direct-Q3 ultrapure water preparation machine.

Text S2 Synthesis and characterization of the catalysts

Urea (20 g) was calcined at 550 °C for 3 h in a muffle furnace at a ramping rate of 5 °C min^{-1}. The synthesized slight yellow powder was denoted as PCN.

A certain amount of PCN intermediates were further calcined at 670 °C in a tube furnace at a ramping rate of 5 °C min^{-1} in an inert atmosphere. Before calcination, the tube furnace was purged by vacuuming and back filling with N_2 for two times. Powders of off-white were afforded after holding them at 670 °C for 2 h and cooling, denoted as C-PCN.

The chemical bonding and crystalline structures of the samples were analyzed using Fourier Transform Infrared (FT-IR) spectroscopy on an IRAffinity-1 infrared spectrometer (Shimadzu, Japan), X-ray Diffraction (XRD) on an ARL EQUINOX 100 X-ray diffractometer (Thermo Fisher Scientific, USA), and X-ray Photoelectron Spectroscopy (XPS) on an ESCALAB 250XI electron spectrometer (VG Scientific, UK). Ultraviolet-visible diffuse reflectance spectroscopy (UV-vis DRS) was conducted using a UV-2550 ultraviolet-visible-near-infrared spectrophotometer (Shimadzu, Japan). Scanning electron microscopy (SEM) was employed to examine the morphology of the catalyst and alkali-treated Providencia bacteria, utilizing a Regulus 8100 field emission scanning electron microscope (Hitachi, Japan). The photoluminescence (PL) and time-resolved photoluminescence (TRPL) spectra were recorded using a Horiba FluoroLog-3 fluorescence spectrometer (Horiba, USA). Reactive species were detected using a Bruker EMX+ electron spin resonance (ESR) spectrometer (Bruker, USA), which was coupled with a F4500 photoluminescence detector (Hitachi, Japan) for signal acquisition. The specific surface area was determined using the Braeuer-Emmett-Teller (BET) method with the Nova 2000e instrument supplied by Quantachrome, USA. The fracture pathway of circular plasmids during the photocatalytic reaction was observed using the APEX-1000 atomic force microscope (AFM) manufactured by Nanjing Emi Instrument Technology Co., Ltd.

Text S3 Detect the antibiotic resistance of *P. alcalifaciens*

The Kirby-Bauer (K-B) disk diffusion method was employed to assess the antibiotic resistance of *P. alcalifaciens* towards to 35 different antibiotics, including erythromycin, penicillin G, and ciprofloxacin. A 100 μL of isolated *P. alcalifaciens* suspension was inoculated into 5 mL of sterile nutrient broth and subsequently was vortexed to ensure homogeneity. The culture was incubated in a temperature-controlled shaking incubator at 37 °C until reaching the logarithmic growth phase. The optical density at 600 nm (OD_{600}) was measured using a UV-Vis spectrophotometer, and the bacterial concentration was adjusted to approximately 10^7 cfu mL^{-1}. The nutrient agar medium was autoclaved for sterilization and then was maintained in a 60 °C water bath for 30 min. Following this, 100 μL of the bacterial suspension was added to 100 mL of melted nutrient agar medium, and the mixture was rapidly and thoroughly vortexed. The solution was then poured into sterile Petri dishes and was allowed to solidify for subsequent use. Within the laminar flow hood, the undersides of the dried plates were labeled with the names of the corresponding antibiotic discs. Sterilized forceps were used to carefully place the antibiotic discs at the designated positions. The inoculated plates were then incubated at 37 °C for 24 h. Following incubation, the antibiotic susceptibility plates were removed, and the diameters of the inhibition zones were measured and recorded. *P. alcalifaciens* exhibited resistance to the following antibiotics: ampicillin, cefathiamidine, cefuroxime, midecamycin, piperacillin, gentamicin, oxacillin, penicillin G, spectinomycin, clarithromycin, vancomycin.

Text S4 Photocatalytic disinfection under visible light irradiation

The experiments of photocatalytic antibacterial were carried out using a PCX-50C multi-channel photochemical reaction system (Beijing Perfectlight Technology Co., Ltd., China). Prior to the experiments, all culture media (nutrient agar, nutrient broth, and PBS), glassware, and pipette tips were sterilized at 121 °C under high pressure for 30 min. *P. alcalifaciens* was inoculated into nutrient broth and was cultured at 37 °C for 12 h. The bacterial culture was then centrifuged and was rinsed with PBS to obtain a standardized bacterial suspension with a concentration of approximately 10^7 cfu mL^{-1}. The experimental temperature was maintained at 30 °C, and the concentration of each added photocatalytic material was set to 0.2 mg mL^{-1}. The visible light intensity was controlled at 100 mW cm^{-2}. For the antibacterial assays, a bacterial suspension of approximately 10^7 cfu mL^{-1} was used. Following the addition of 4 mg of catalyst powder to 10 mL of PBS containing *P. alcalifaciens*, the reaction was initiated. At specified irradiation time intervals, 0.1 mL of the treated bacterial suspension was collected, was serially diluted with PBS, and was spread onto nutrient agar plates. The plates were incubated at 37 °C for 20 h, after which the CFUs were counted to assess bacterial viability.

Text S5 Sample preparation for SEM analysis of *P.alcalifaciens*

The morphological changes of *P. alcalifaciens* cells before and after inactivation treatment were characterized by SEM. Under visible light irradiation, the catalyst C-PCN (0.4 mg mL^{-1}) was added to the PBS solution (10 mL, with a bacterial concentration of 10^7 cfu mL^{-1}) containing *P. alcalifaciens* bacteria and was reacted for 100 min. Meanwhile, a control group without the catalyst was set up. Every 20 min, 1 mL of the bacterial solution was taken out, washed three times with PBS (each time for 3-5 min), and centrifuged at 8000 rpm for 3 min at 4°C. The samples were then fixed with 2.5% glutaraldehyde at 4°C for 8-24 h and were centrifuged at 8000 rpm for another 3 min at 4°C. The supernatant was discarded, and the samples were washed three times with PBS (each time for 3-5 min). The bacteria were dehydrated with anhydrous ethanol concentrations of 30%, 50%, 70%, 90%, and finally, were observed by SEM.

Text S6 Sample preparation for *P.alcalifaciens* viability assessment using the Calcein/PI assay

Under visible light irradiation, the catalyst C-PCN (0.4 mg mL^{-1}) was added to the PBS solution (10 mL, with a bacterial concentration of 10^7 cfu mL^{-1}) containing *P. alcalifaciens* bacteria and was reacted for 100 min. Meanwhile, a control group without the catalyst was set up. At 20 min, 1 mL aliquots of the bacterial suspension were withdrawn and diluted with PBS to obtain 100 μL samples maintaining a bacterial concentration of 10^7 cfu mL^{-1}. The treated bacterial solution samples were centrifuged at 12,000 rpm for 1 min at 4°C; then, 10 μL of a 0.85% NaCl solution was added and mixed well by shaking before adding 0.3 μL of the Calcein-AM/PI mixed solution was added. The samples were incubated in the dark at room temperature for 15 min. Then, 10 μL of the sample was dropped onto a glass slide; a cover slip was placed on top, and *P. alcalifaciens* was imaged using a high-resolution laser confocal microscope (ZEISS, LSM 880).

Text S7 Bacterial regrowth experiment

Studies on the resuscitation capacity of bacteria had demonstrated that certain bacterial species could enter a viable but non-culturable state during or following water disinfection processes. In this physiological state, the bacteria remained metabolically active but were unable to form colonies on conventional culture media. Despite this limitation, they retained the potential to regenerate and reactivate during the storage and distribution of treated water. Therefore, it was essential to evaluate the regrowth potential of bacteria in photocatalytic disinfection water. Bacterial samples collected at various reaction time were stored in a 4 °C refrigerator for 24 h to induce dormancy. Subsequently, 5 mL of nutrient broth was added to each sample, and the suspensions were incubated in a 37 °C constant-temperature shaker for different time (24 and 48 h). The presence of viable target bacterial cells was then assessed using nutrient agar solid medium.

Text S8 Cyclic stability assessments

The long-term stability and antibacterial performance of biomaterials were critical factors for their practical applications. To evaluate the long-term stability and multi-cycle contact antibacterial activity of C-PCN, the residual solution obtained after a single antibacterial kinetics reaction was sterilized, filtered, and centrifuged to recover the catalyst. The recovered catalyst was then dried at 60 °C and was reused in subsequent antibacterial tests. This process was repeated five times, and the antibacterial efficacy was assessed using the standard plate count method.

Text S9 Protein leakage and conductivity assays

The bacterial apoptosis mechanism was investigated by measuring the protein concentration and conductivity of the bacterial suspension following photocatalytic treatment. In this study, C-PCN catalyst (0.4 mg mL^{-1}) was introduced into a PBS solution (10 mL) containing *P. alcalifaciens* at a concentration of 10^7 cfu mL^{-1}, and the reaction was carried out under visible light irradiation for 100 min. A control group without the addition of catalyst was also established for comparison. At 20-min intervals, 1 mL of the reaction mixture was sampled to measure conductivity using a conductivity meter. The remaining portion of each sample was centrifuged at 4 °C and 12,000 × g for 2 min, and the supernatant was transferred to a 96-well microplate. The protein concentration in the supernatant was then quantified at an optical density of 562 nm (OD_{562}) using an enhanced BCA protein assay kit on an enzyme-linked immunosorbent assay (ELISA) plate reader.

Text S10 Lipid oxidation detection

Malondialdehyde (MDA) was a naturally occurring byproduct of lipid peroxidation in biological systems. When oxidative stress occurred in animal or plant cells, lipid peroxidation was initiated. During this process, certain fatty acids underwent gradual oxidation and decomposed into a series of complex compounds, among which MDA was a representative marker. Therefore, the extent of lipid peroxidation could be assessed by measuring MDA levels, and MDA quantification was widely used as a biochemical indicator of oxidative damage. In this study, under visible light irradiation, C-PCN catalyst (0.4 mg mL^{-1}) was introduced into a PBS solution (10 mL) containing *P. alcalifaciens* at a concentration of 10^7 cfu mL^{-1}, and the reaction was carried out for 100 min. A control group without the addition of the catalyst was also included. At 20 min intervals, 1 mL of the bacterial suspension was collected. Subsequently, 100 μL of IP cell lysis buffer (P0013) was added to each sample, followed by centrifugation at 12,000 × g for 10 min at 4 °C. The concentration of MDA in the supernatant was then determined at an optical density of 532 nm (OD_{532}) using an MDA lipid peroxidation assay kit on a microplate reader.

Text S11 Alkaline phosphatase activity assay

Alkaline phosphatase (AKP) was an enzyme that catalyzes the hydrolysis of phosphate ester bonds under alkaline conditions.

Under alkaline conditions, 4-methylumbelliferyl phosphate disodium salt (4-MUP) was hydrolyzed by alkaline phosphatase into 4-methylumbelliferone (4-MU) and inorganic phosphate. The resulting 4-MU emitted blue fluorescence with an excitation wavelength of 360 nm and an emission wavelength of 450 nm. The fluorescence intensity of 4-MU was directly proportional to the amount of substrate converted by alkaline phosphatase, allowing the enzyme activity to be quantitatively determined based on fluorescence measurements.

In this experiment, under visible light irradiation, C-PCN catalyst (0.4 mg mL^{-1}) was introduced into a PBS solution (10 mL) containing *P. alcalifaciens* at a concentration of 10^7 cfu mL^{-1}, and the reaction was carried out for 100 min. A control group without the addition of the catalyst was also included for comparison. At 20 min intervals, 1 mL of the bacterial suspension was collected. The cells were pelleted via centrifugation (e.g., 100-500 × g, 5 min), and the supernatant was carefully removed. BeyoLysis™ Buffer A for Metabolic Assay (100-200 µL per 1 million cells) was added, followed by gentle pipetting and incubation on ice for 5-10 min to ensure complete cell lysis. The lysate was then centrifuged at approximately 12,000 × g for 3-5 min at 4 °C, and the supernatant was collected for fluorescence analysis. Fluorescence detection was performed using an enzyme-linked immunosorbent assay (ELISA) plate reader with excitation and emission wavelengths of 360 nm and 450 nm, respectively.

Text S12 PCR and gel electrophoresis experiments

The polymerase chain reaction (PCR) reaction mixture contained 10 μL of One Taq Quick-Load 2× Master Mix, 1 μL of each forward and reverse primer, 3 μL of template DNA, and dd-H_2O to a final volume of 20 μL. Amplification followed thermal cycling conditions: initial denaturation at 94 °C for 5 min, followed by 30 cycles of denaturation at 94 °C for 45 s, annealing at 55 °C for 45 s, and extension at 72 °C for 1 min, with a final extension step at 72 °C for 5 min. The resulting PCR products were resolved on a 1.5% agarose gel and visualized using a gel documentation system.

Text S13 qPCR detection of target ARGs

Firstly, bacterial suspensions were collected at designated time intervals, and cells were harvested via centrifugation. Then, intracellular DNA was isolated from the resulting cell pellets using the Ultra Clean DNA Isolation Kit (MoBiO Laboratories, USA), following the instructions of manufacturer. Extracellular DNA was treated with lysozyme, RNase A, and proteinase K, followed by purification and concentration using a DNA purification column (Shandong, China).

The setting of amplification conditions for fluorescence quantitative PCR: volume and final concentration.

Amplification curve: 94℃ for 30 s; 94℃ for 10 s, 60℃ for 12 s, and 72℃ for 30 s for a total of 45 cycles, with single-point detection signal at 72℃.

Melting curve: 95℃ for 0 s, then to 65℃ for 15 s, back to 95℃ for another 0 s, with continuous detection signal.

Text S14 AFM image of plasmid

Initially, TE solution (10 mmol L^{-1} Tris-HCl + 1 mmol L^{-1} EDTA, pH 8.0) was supplemented with $MgCl_2$ to achieve a final $MgCl_2$ concentration of 3.5 mmol L^{-1}. The original plasmid solution was then diluted to a concentration of 1 ng μL^{-1} using the TE buffer containing 3.5 mmol L^{-1} $MgCl_2$. Subsequently, a clean mica surface was prepared by cleaving a mica sheet. Approximately 10 μL of the diluted plasmid solution was deposited onto the mica surface using a micropipette and was allowed to adsorb for 3 min. The sample was then rinsed with approximately 200 mL of ultrapure water and was dried under a stream of nitrogen gas before being placed in a desiccator for at least 1 h to ensure complete drying prior to scanning. Based on the random distribution of plasmids, the surface morphology of each sample was analyzed within selected fields of view, and the predominant imaging patterns were used to determine the final structural state of the plasmids.

Text S15 Photoelectrochemical measurements

Photoelectrochemical measurements were conducted on the CHI660D electrochemical workstation equipped with a three-electrode system to measure the photocurrent response and EIS using a platinum wire electrode, saturated calomel electrode, and working electrode. Using 0.5 M Na_2SO_4 solution as the electrolyte, the preparation method of the working electrode was as follows: 5 mg of the sample powder was ultrasonically dispersed in 1 mL of ultrapure water, coated on the surface of a 20×40 mm indium tin oxide glass, was dried overnight at room temperature, and then was heated in an oven at 180 °C for 5 h. The photocurrent response test was conducted at 0.0 V, and the EIS measurement was carried out within the frequency range of 0.01 to 10.5 Hz.

An Ag/AgCl electrode (saturated KCl) was employed as the reference electrode, and a platinum wire served as the counter electrode. A glassy carbon electrode was used as the working electrode for EIS measurements. The transient photocurrent response was recorded in 0.5 M Na_2SO_4 solution under an applied bias voltage of ±1.0 V via I-T curve analysis. The EIS results were acquired by applying a 5 mV AC voltage amplitude in 0.5 M Na_2SO_4 solution across a frequency range from 50 kHz to 1 kHz.

Text S16 Biological toxicity analysis

WST-8, a component of the CCK-8 reagent, was reduced by mitochondrial dehydrogenases in living cells to form a yellow-colored formazan product, the concentration of which was directly proportional to the number of viable cells. The absorbance at 450 nm could be measured using an enzyme-linked immunosorbent assay (ELISA) plate reader to quantitatively assess cell viability. The cytotoxicity of various concentrations of C-PCN (0.2 mg mL^{-1}, 0.4 mg mL^{-1}, 0.6 mg mL^{-1} and 0.8 mg mL^{-1}) on NIH/3T3 cells was evaluated using the CCK-8 assay.

The culture medium was prepared by supplementing Dulbecco's Modified Eagle Medium (DMEM) with 10% fetal bovine serum (FBS), 1% penicillin-streptomycin solution (HyClone), and 89% liquid DMEM. NIH/3T3 cells were cultured in this complete medium at 37 °C in a humidified incubator with 5% CO_2. The culture medium was refreshed daily. When the cells reached the logarithmic growth phase, they were seeded into a 96-well microplate at a density of 5×10^4 cells mL^{-1} in 100 μL of fresh DMEM per well, with each condition tested in duplicate. After cell attachment, the supernatant was removed, and 100 μL of C-PCN solutions at different concentrations were added to the respective wells. The cells were then co-cultured with the test materials at 37 °C for 4 h. Subsequently, 10 μL of CCK-8 reagent was added to each well, and the plate was further incubated at 37 °C for 4 h. Finally, the absorbance at 450 nm was measured using an ELISA plate reader. The sample without C-PCN treatment served as the negative control group.

Text S17 Antibacterial for flowing water bodies

The reactor had a total volume of 3 L, with a catalyst concentration of 0.4 g L^{-1} and a flow rate of 1.5 L h^{-1}. Under sunlight irradiation, flowing hospital wastewater was subjected to disinfection, and the bacterial counts as well as the residual levels of antibiotic resistance genes were measured at the outlet.

Text S18. ROS fluorescence detection

We evaluated the generation of ROS by *P. alcalifaciens* at various time intervals during the photocatalytic process in the presence of 0.4 mg mL^{-1} C-PCN (Fig. S6). This approach enabled a relatively quantitative assessment of the antibacterial activity. The experimental results demonstrated that ROS production increased progressively with prolonged reaction time, which aligned well with the observed antibacterial efficacy.

Figures

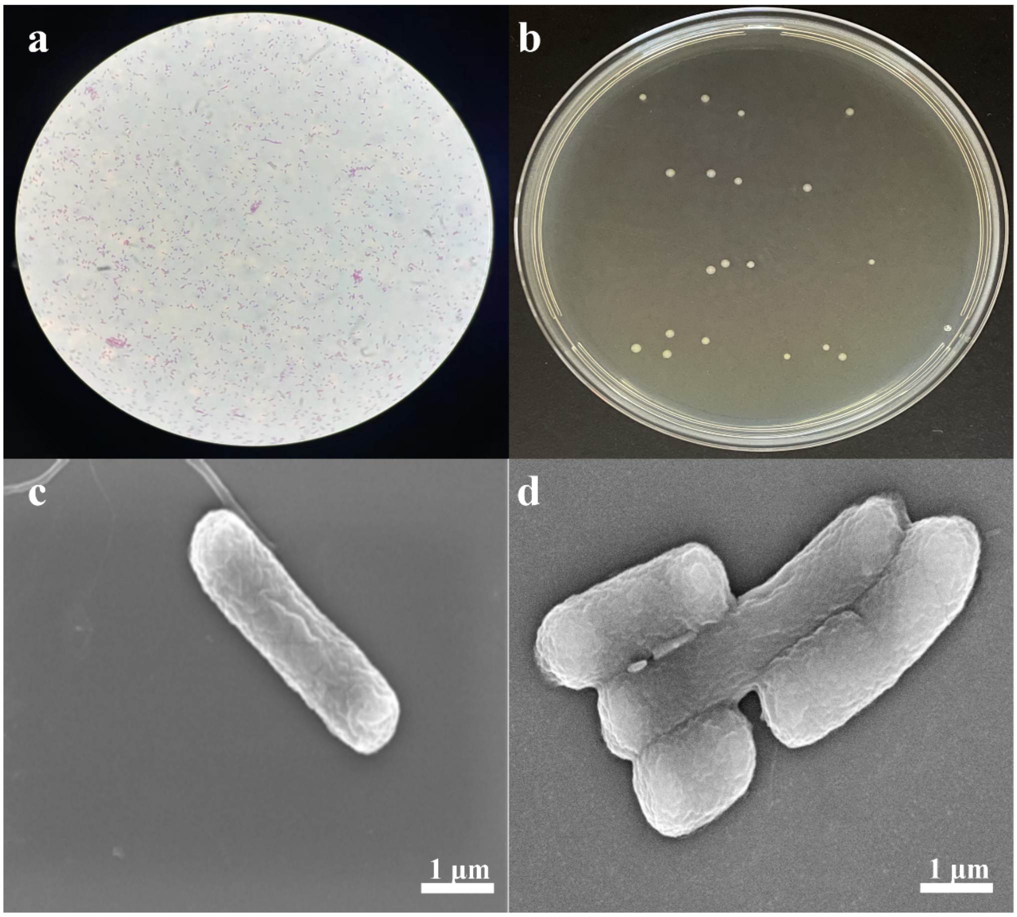

Fig. S1 Morphological and structural characteristics of *P. alcalifaciens* (a-b) microscopic image of *P. alcalifaciens*, colony plate map, (c-d) SEM images of *P. alcalifaciens*.

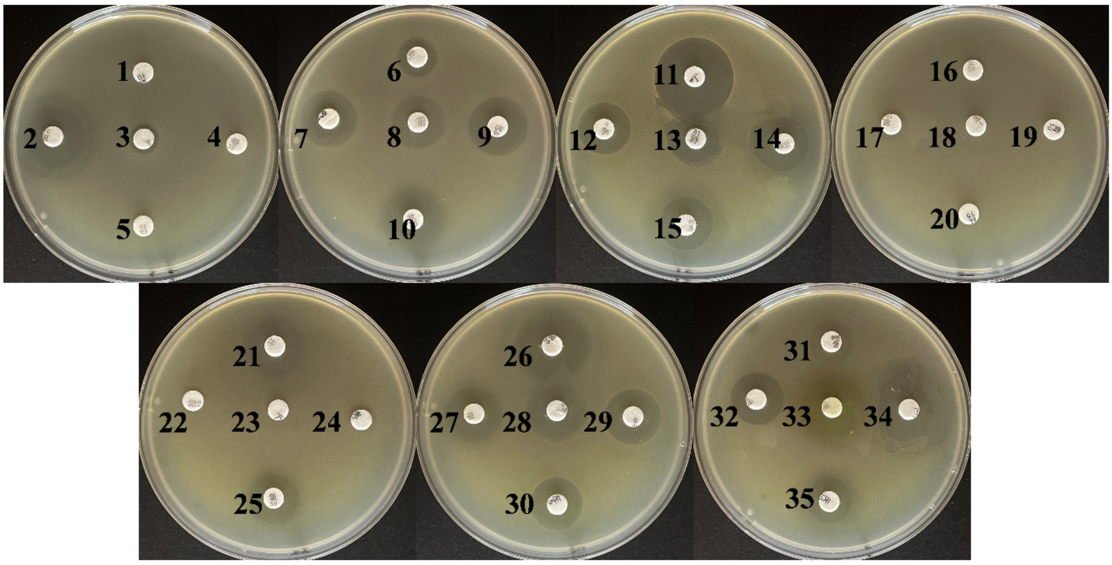

Fig. S2 Antimicrobial susceptibility assay of *P. alcalifaciens*.

(1) Ampicillin, (2) Aztreonam, (3) Cefazolin, (4) Cefathiamidine, (5) Cefuroxime, (6) Cefoperazone, (7) Cefotaxime, (8) Ceftriaxone, (9) Cefepime, (10) Ceftazidime, (11) Cefoxitin, (12) Ofloxacin, (13) Streptomycin, (14) Tobramycin, (15) Kanamycin, (16) Midecamycin, (17) Piperacillin, (18) Gentamicin, (19) Oxacillin, (20) Penicillin G, (21) Erythromycin, (22) Spectinomycin, (23) Clarithromycin, (24) Vancomycin, (25) Clindamycin, (26) Chloramphenicol, (27) Heminiksin, (28) Tetracycline, (29) Minocycline, (30) Norfloxacin, (31) Ciprofloxacin, (32) Levofloxacin, (33) Polymyxin B, (34) Compound Sulfamethoxazole, (35) Nitrofurantoin.

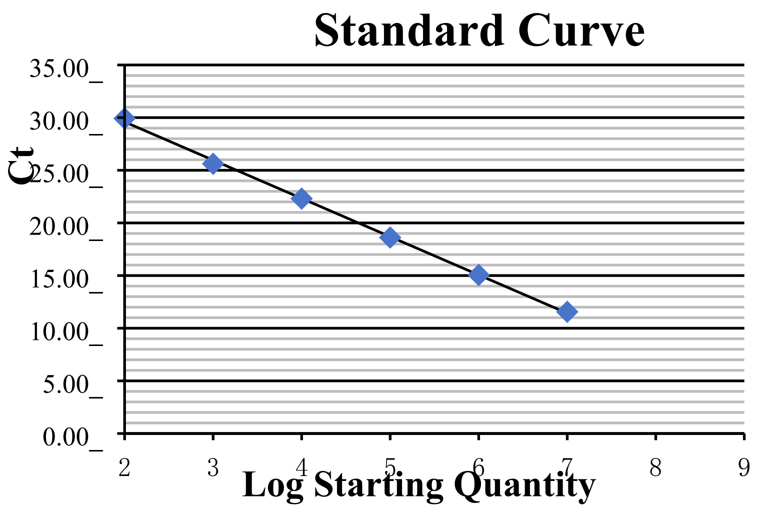

Fig. S3 Absolute quantitative standard curve for QnrS2 gene qPCR.

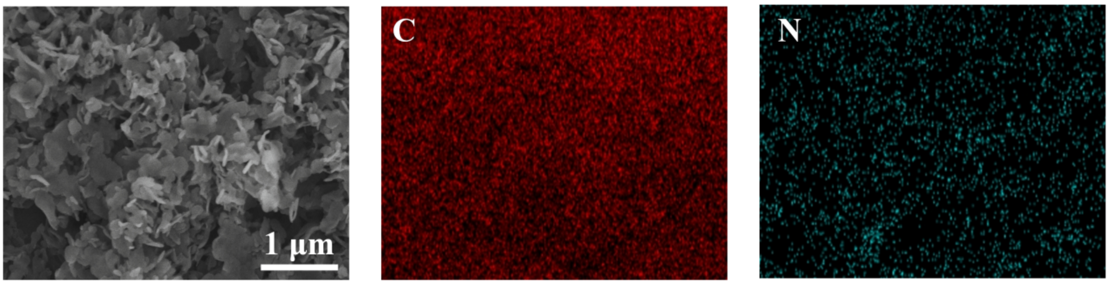

Fig. S4 Elemental mapping of C (red), and N (blue) in the C-PCN.

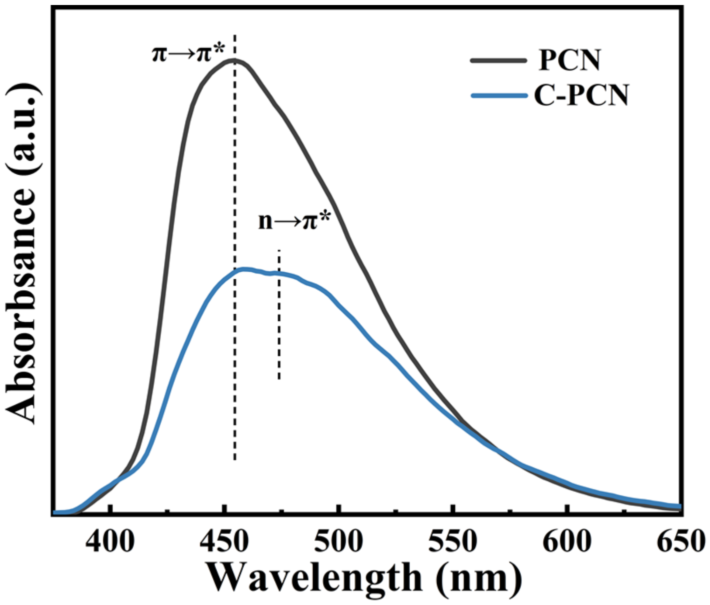

Fig. S5 PL spectra of PCN and C-PCN samples.

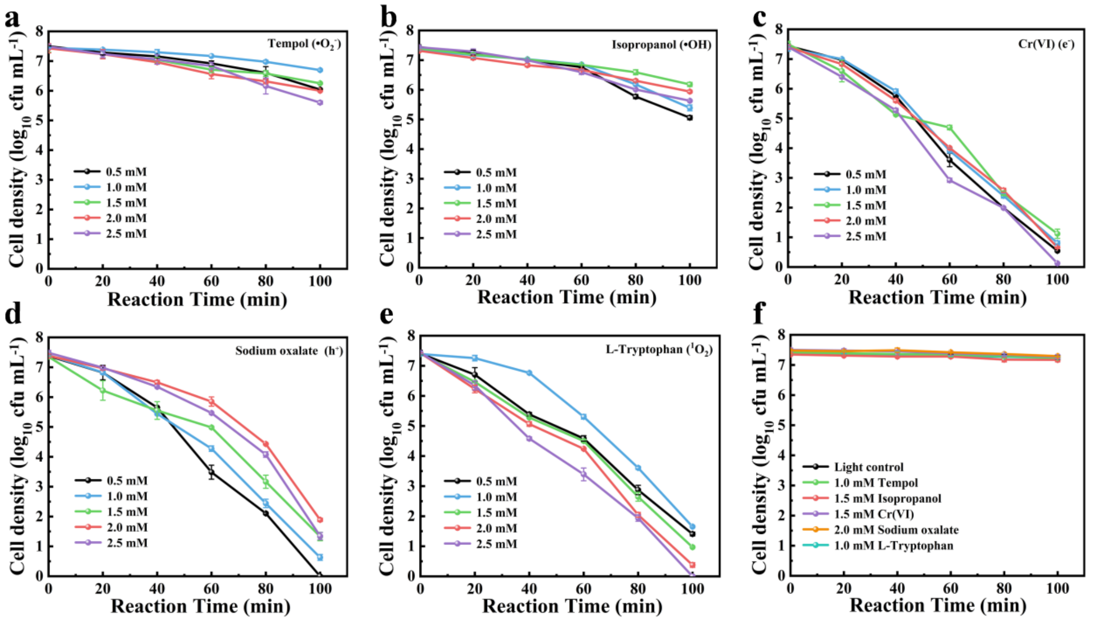

Fig. S6 (a) The experiments on the capture of $\cdot O_2^-$ by tempol of different concentrations, (b) the experiments on the capture of $\cdot OH$ by isopropanol of different concentrations, (c) the experiments on the capture of h^+ by sodium oxalate of different concentrations, (d) the experiments on the capture of $^1O_2$ by L-tryptophan of different concentrations, (e) the experiments on the capture of e^- by Cr (VI) of different concentrations, (f) toxicity experiments of different capture agents on bacteria.

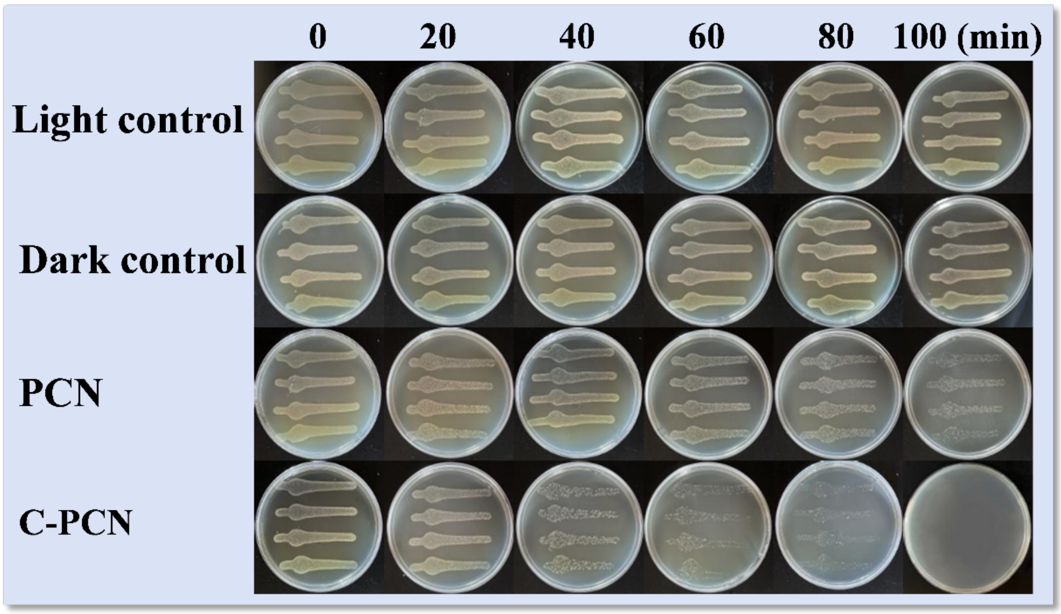

Fig. S7 The colony plates of photocatalytic disinfection experiments.

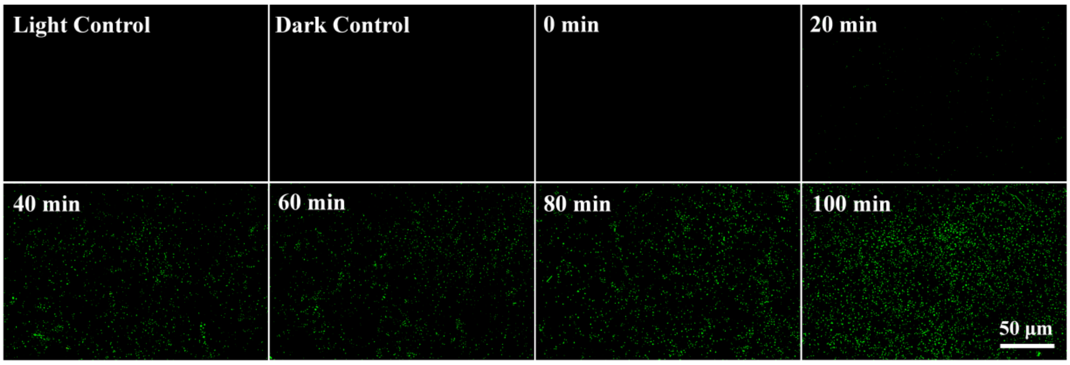

Fig. S8 ROS fluorescence detection.

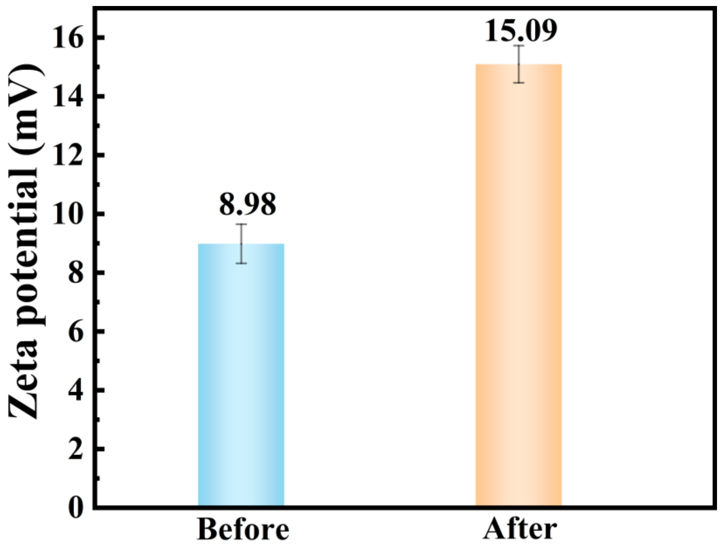

Fig. S9 Zeta tests on the *P. alcalifaciens* before and after the photocatalytic antibacterial.

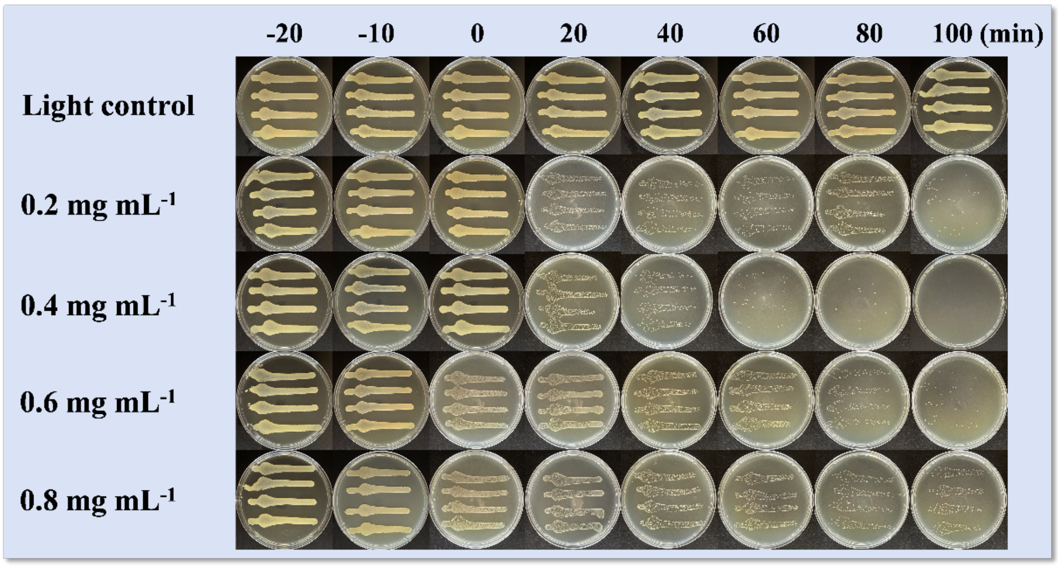

Fig. S10 Antibacterial experiments with different catalyst concentrations.

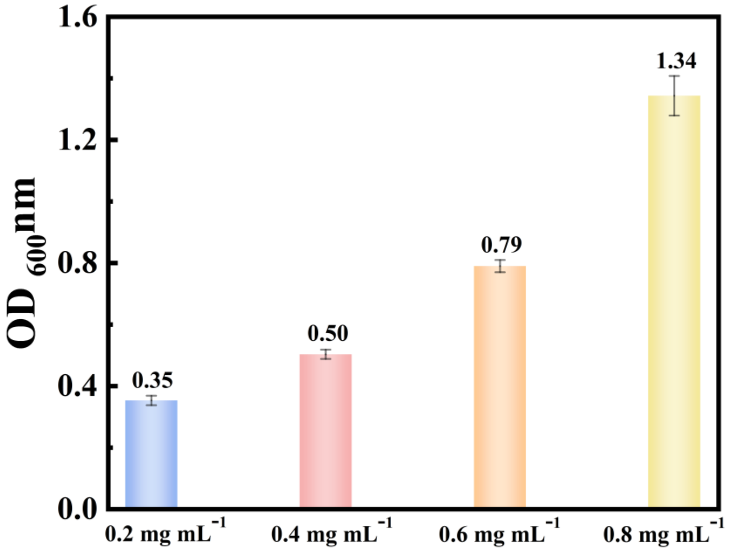

Fig. S11 The absorbance of bacterial suspensions with different material concentrations at 600 nm.

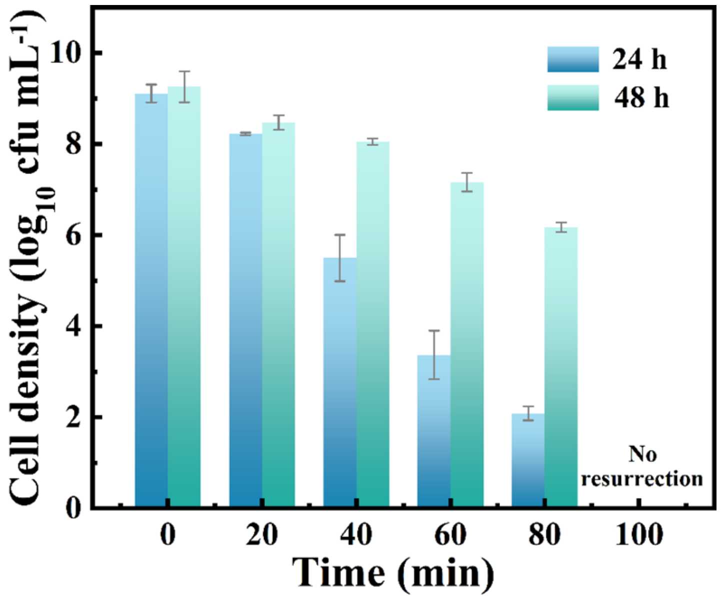

Fig. S12 Antibacterial experiments under different bacterial concentrations.

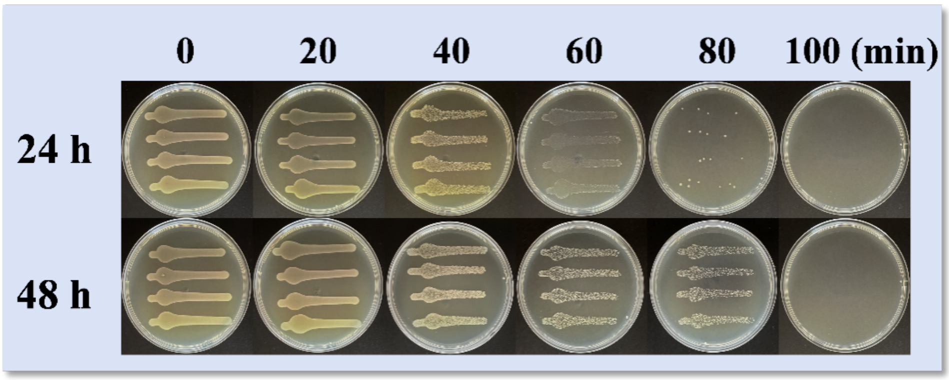

Fig. S13 Photographs of bacterial agar plates for bacterial recovery.

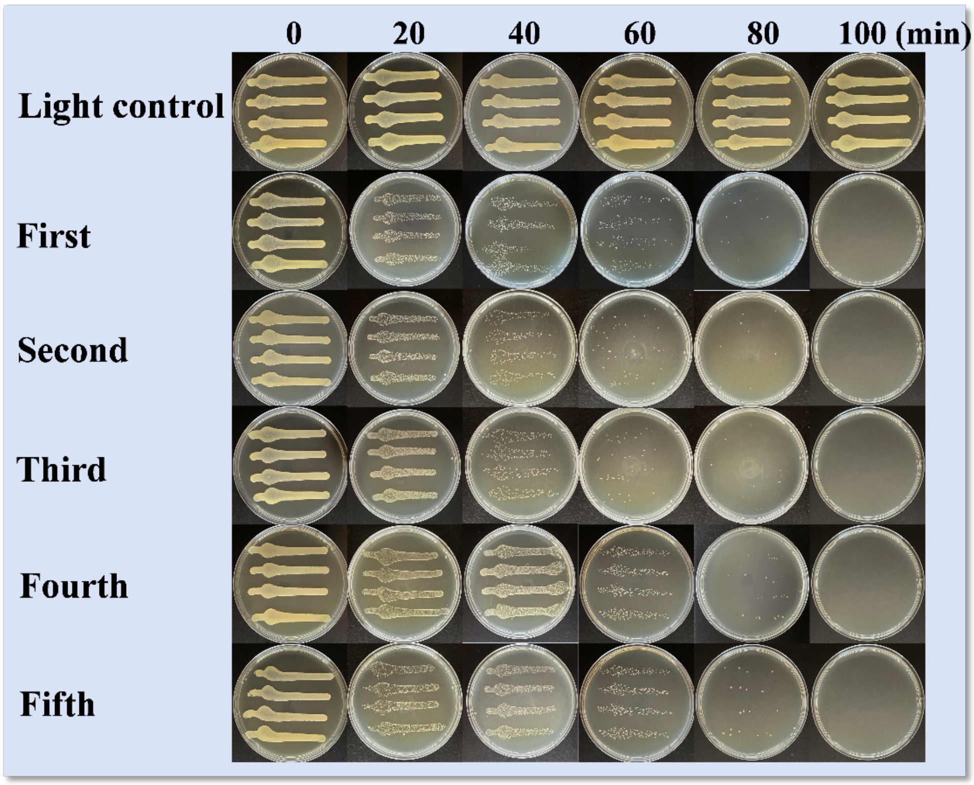

Fig. S14 Antibacterial cyclic stability colony plate.

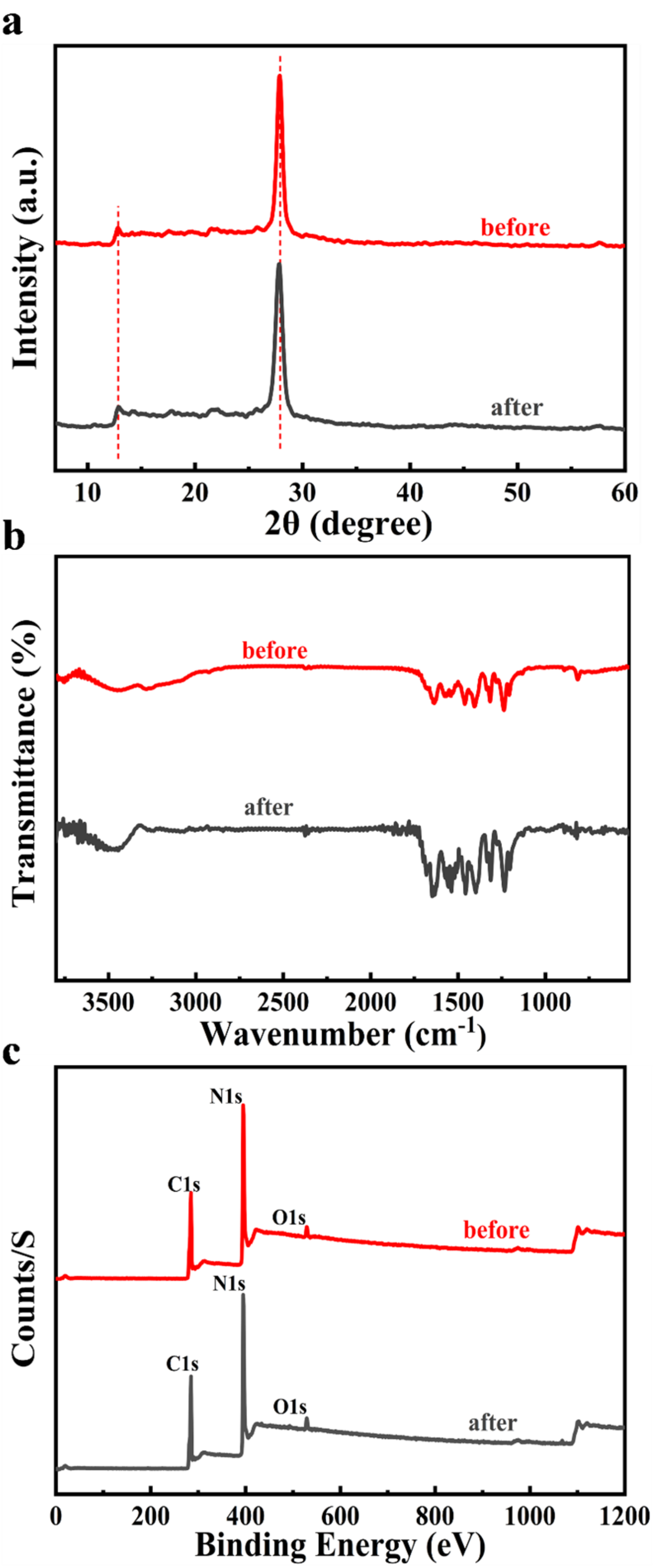

Fig. S15 Characterization of the cyclic stability of C-PCN: (a) XRD patterns of C-PCN before and after the cycling test, (b) FT-IR spectra of C-PCN before and after the cycling test, (c) XPS survey spectrum spectra of C-PCN before and after the cycling test.

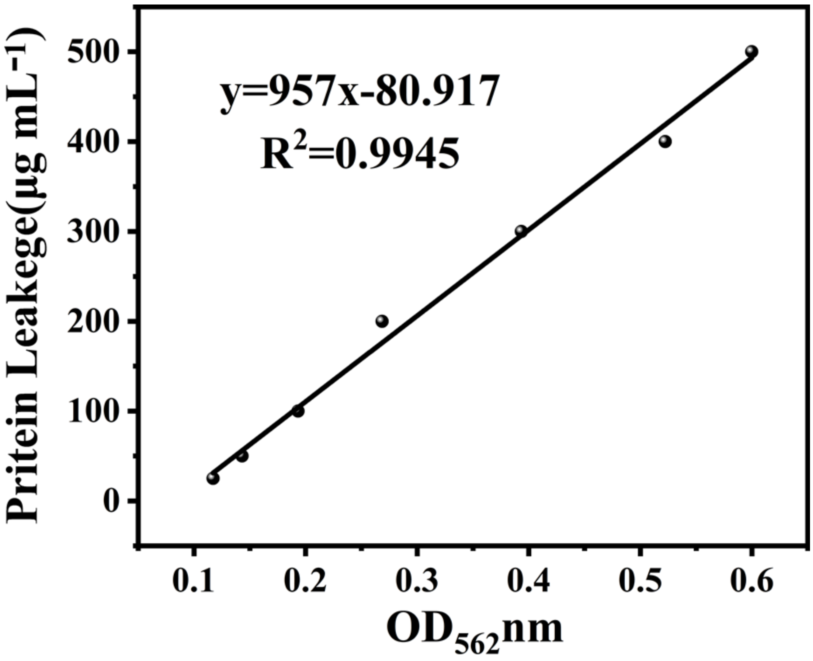

Fig. S16 Standard protein concentration vs. standard curve of OD562 nm.

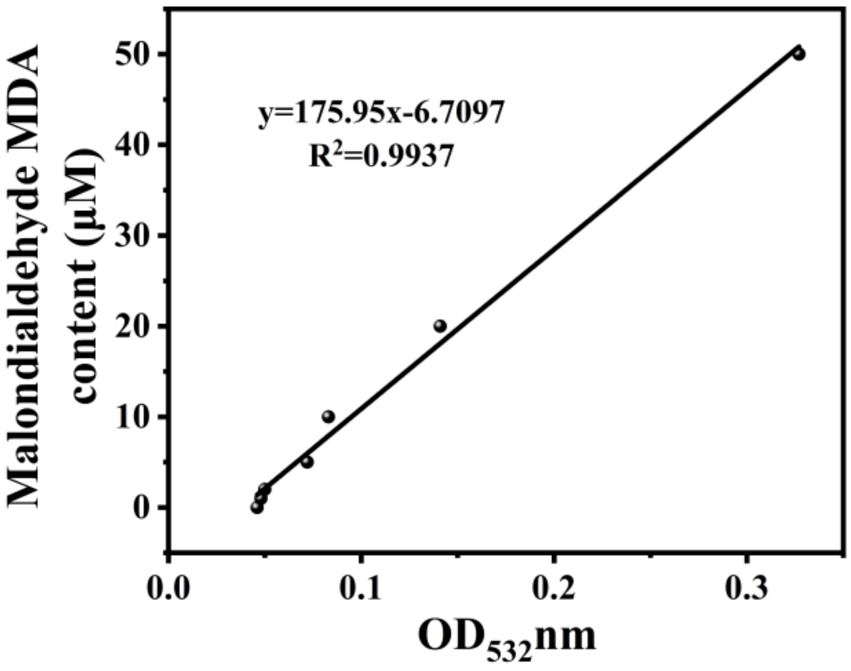

Fig. S17 Standard MDA concentration vs. standard curve of OD532 nm.

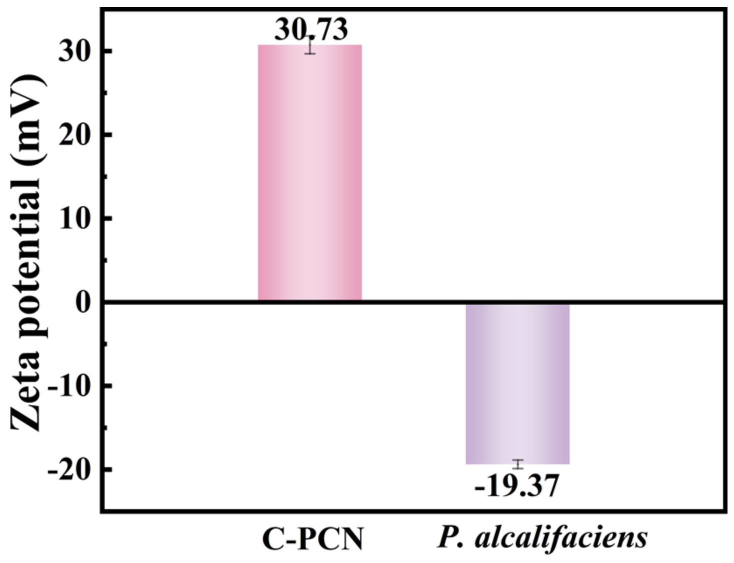

Fig. S18 Zeta tests of the C-PCN and *P. alcalifaciens*.

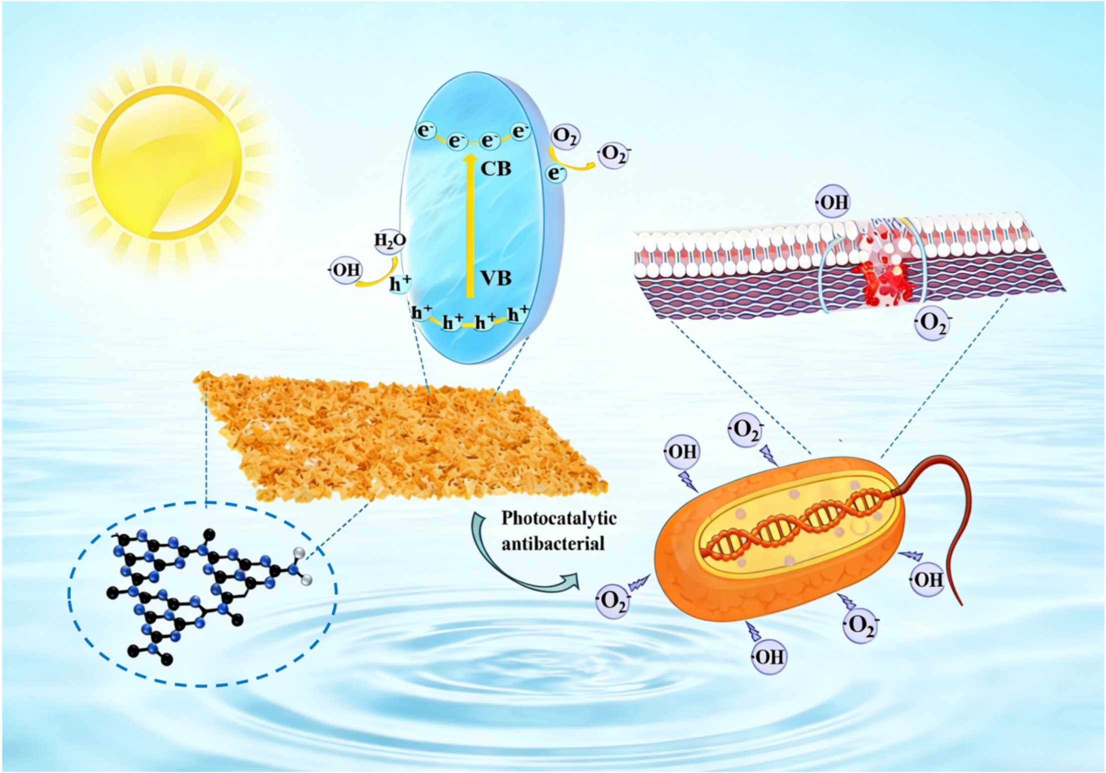

Fig. S19 The antibacterial application mechanism of nanocomposites in addressing multi-drug resistance

Tables

Table S1 Identification of strains

Description	Max Score	Total Score	Query Cover	E value	Per. Ident	Accession
Providencia alcalifaciens strain 2019-04-29291-1-1 genome assembly, chromosome: 1	2728	19100	100%	0.0	100.00%	OU659204.1
Providencia rustigianii strain NCTC8113 genome assembly, chromosome: 1	2717	18978	100%	0.0	99.86%	LR134396.1
Providencia alcalifaciens strain HXM20 16S ribosomal RNA gene, partial sequence	2717	2717	100%	0.0	99.86%	MZ734420.1
Providencia rustigianii strain NCTC6933 genome assembly, chromosome: 1	2712	18928	100%	0.0	99.80%	LR134189.1
Providencia alcalifaciens strain NCTC10286 genome assembly, chromosome: 1	2712	18952	100%	0.0	99.80%	LS483467.1
Providencia alcalifaciens strain FDAARGOS_408 chromosome, complete genome	2712	18919	100%	0.0	99.80%	CP023536.1
Providencia alcalifaciens strain 205/92 chromosome, complete genome	2712	18950	100%	0.0	99.80%	CP151776.1
Providencia zhijiangensis strain D4759 chromosome, complete genome	2712	18984	100%	0.0	99.80%	CP135990.1
Providencia alcalifaciens strain 2939/90 chromosome, complete genome	2712	18950	100%	0.0	99.80%	CP116943.1
Providencia alcalifaciens strain LHC2-1 chromosome, complete genome	2712	18967	100%	0.0	99.80%	CP084296.1

Table S2 Primer sequences of QnrS2

Primer	Sequence (5' to 3')	Number of bases	Purification method	Bp size
QnrS2-F	CACACATATCGACACCAC	18	PAGE	189
QnrS2-R	CTTGCATCGCGAAGGTCT	18	PAGE	

Table S3 FQ-PCR reaction system

Components	Volume	Final concentration
SYBR Premix Ex Taq (2×)	6 μL	1×
Forward primers (10 μM)	0.3 μL	0.4 μM
Reverse primers (10 μM)	0.3 μL	0.4 μM
Template/genomic DNA	1 μL	
ddH_2O	to 12 μL	

Table S4 Absolute quantification standard curve concentration of qPCR for QnrS2 gene

Standard	Ct1	Ct2	Ct3	Mean	SD
$1*10^7$	11.68	11.56	11.43	11.56	0.13
$1*10^6$	14.88	15.02	15.27	15.06	0.20
$1*10^5$	18.65	18.62	18.57	18.61	0.04
$1*10^4$	22.22	22.32	22.38	22.31	0.08
$1*10^3$	25.58	25.61	25.65	25.61	0.04
$1*10^2$	29.78	30.15	29.84	29.92	0.20

Table S5 The BET of PCN and C-PCN: surface area, pore volume and average pore diameter

Photocatalysts	BET Surface Area (m^2 g^{-1})	Pore Volume (cm^3 g^{-1})	Average Pore Size (nm)
PCN	79.9	0.5	23.2
C-PCN	84.1	0.7	26.9

Table S6 Quantitative analysis of element content

Photocatalysts	C atomic conc (%)	N atomic conc (%)
PCN	47.96	50.07
C-PCN	48.05	50.01
